# Supplementary material for: Concurrent Disorder Management Guidelines. Systematic Review
Source: J Clin Med. 2020 Jul 28;9(8):2406. doi: 10.3390/jcm9082406 (PMC7463987; doi:10.3390/jcm9082406)
Supplement: Supplementary file 1 [file jcm-09-02406-s001.pdf]

Select Resource

UNIV OF BRITISH COLUMBIA

EBSCOhost Web
PsycINFO
PsycINFO
Primary search
Demo Search Builder
CINAHL With Full Text
Pre-CINAHL
CINAHL With Full Text Search Builder
Biomedical Reference Collection
EBSCO Music Databases
Business Source Complete

Business Searching Interface

EBSCOhost Image Searching

Images from Journal Articles in EBSCO Databases

Medical Images from CHC

Native American Archives

EBSCO Discovery Service

WilsonWeb Indexes

DynaMed

Health Databases

Explora Primary Schools

Explora Secondary Schools

Explora Educator's Edition

DynaMed Plus

EBSCO Support Site Privacy Policy Terms of Use Copyright

© 2020 EBSCO Industries, Inc. All rights reserved

Search History/Alerts

Print Search History | Retrieve Searches | Retrieve Alerts | Save Searches / Alerts

Select / deselect all | Refresh Search Results

Search with AND | Search with OR | Delete Searches

Search ID#	Search Terms	Search Options	Actions
S15	S11 AND S14	**Search modes -** Boolean/Phrase	Rerun View Details Edit
S14	S12 OR S13	**Search modes -** Boolean/Phrase	Rerun View Details Edit
S13	(Guideline* or consensus or best practice or recommendation*)	**Search modes -** Boolean/Phrase	Rerun View Details Edit
S12	(MH "Practice Guidelines")	**Search modes -** Boolean/Phrase	Rerun View Details Edit
S11	S9 OR S10	**Search modes -** Boolean/Phrase	Rerun View Details Edit
S10	S7 AND S8	**Search modes -**	Rerun

			Boolean/Phrase	View Details<br>Edit
☐	S9	S4 AND S8	**Search modes** - Boolean/Phrase	Rerun<br>View Details<br>Edit
☐	S8	(concurrent or dual diagnos* or co-occurring or comorbidity or co-existing or coinciding)	**Search modes** - Boolean/Phrase	Rerun<br>View Details<br>Edit
☐	S7	S5 OR S6	**Search modes** - Boolean/Phrase	Rerun<br>View Details<br>Edit
☐	S6	("drug abuse" or "drug addiction" or "substance abuse" or alcohol or tobacco or nicotine or marijuana or cannabis or heroin or stimulant* or crystal meth or methamphetamine or phencyclidine or MDMA or GHP or "club drug*" or LSD or depressant* or stimulants or hallucinogen* or	**Search modes** - Boolean/Phrase	Rerun<br>View Details<br>Edit

		"prescription narcotic*")		
	S5	(MH "Substance Abuse+") OR (MH "Substance Dependence+") OR (MH "Alcohol-Related Disorders+") OR (MH "Inhalant Abuse") OR (MH "Substance Abuse, Intravenous") OR (MH "Smoking") OR (MH "Substance Withdrawal Syndrome+")	**Search modes** - Boolean/Phrase	Rerun View Details Edit
	S4	S1 OR S2 OR S3	**Search modes** - Boolean/Phrase	Rerun View Details Edit
	S3	(insomnia or depression or mood or bipolar or mania or anxiety or post traumatic stress disorder* or psychosis or schizo*)	**Search modes** - Boolean/Phrase	Rerun View Details Edit
	S2	2 ((psychiatric	**Search modes** -	Rerun

		or mental or behaviou#r or anxiety or bipolar or dissociative or mood) N3 (disorder* or diagnosis* or illness* or disease*))	Boolean/Phrase	View Details<br>Edit
☐	S1	(MH "Mental Disorders+") OR (MH "Adjustment Disorders+") OR (MH "Mental Disorders, Chronic") OR (MH "Neurotic Disorders+") OR (MH "Personality Disorders+") OR (MH "Psychotic Disorders+") OR (MH "Sexual and Gender Disorders+") OR (MH "Psychiatric Emergencies") OR (MH "Psychological Trauma")	**Search modes -** SmartText Searching	Rerun<br>View Details<br>Edit

Brought to you by The University of British Columbia Library.

powered by EBSCO*host*

© 2020 EBSCO Industries, Inc. All rights reserved.

Search History (17)

# ▲	Searches	Results	Type	Actions	Annotations
1	mental disorders/ or exp anxiety disorders/ or exp "bipolar and related disorders"/ or exp "disruptive, impulse control, and conduct disorders"/ or exp dissociative disorders/ or exp elimination disorders/ or exp "feeding and eating disorders"/ or exp mood disorders/ or exp neurotic disorders/ or exp paraphilic disorders/ or exp personality disorders/ or exp "schizophrenia spectrum and other psychotic disorders"/ or exp sexual dysfunctions, psychological/ or exp sleep wake disorders/ or exp somatoform disorders/ or exp "trauma and stressor related disorders"/	685388	Advanced	Display Results | More	
2	((psychiatric or mental or behaviou?r or anxiety or bipolar or dissociative or mood) adj3 (disorder* or diagnos#s or illness* or disease*)).mp. [mp=title, abstract, original title, name of substance word, subject heading word, floating sub-heading word, keyword heading word, organism supplementary concept word, protocol supplementary concept word, rare disease supplementary concept word, unique identifier, synonyms]	361755	Advanced	Display Results | More	
3	(insomnia or depression or mood or bipolar or mania or anxiety or post traumatic stress disorder* or psychosis or schizo*).mp. [mp=title, abstract, original title, name of substance word, subject heading word, floating sub-heading word, keyword heading word, organism supplementary concept word, protocol supplementary concept word, rare disease supplementary concept word, unique identifier, synonyms]	795706	Advanced	Display Results | More	
4	1 or 2 or 3	1194303	Advanced	Display Results | More	
5	substance-related disorders/ or exp alcohol-related disorders/ or exp amphetamine-related disorders/ or exp cocaine-related disorders/ or exp drug overdose/ or exp inhalant abuse/ or exp marijuana abuse/ or exp narcotic-related disorders/ or exp phencyclidine abuse/ or exp psychoses, substance-induced/ or exp substance abuse, intravenous/ or exp substance abuse, oral/ or exp substance withdrawal syndrome/ or exp "tobacco use disorder"/	273431	Advanced	Display Results | More	
6	(drug or substance or alcohol or tobacco or nicotine or marijuana or cannabis or heroin or stimulant or crystal meth or methamphetamine or phencyclidine or MDMA or GHP or club drugs or LSD or depressant* or stimulant* or hallucinogen* or prescription narcotic*).mp. [mp=title, abstract, original title, name of substance word, subject heading word, floating sub-heading word, keyword heading word, organism supplementary concept word, protocol supplementary concept word, rare disease supplementary concept word, unique identifier, synonyms]	6053867	Advanced	Display Results | More	
7	5 or 6	6091065	Advanced	Display Results | More	
8	(concurrent or dual diagnos* or co-occurring or comorbidity or co-existing or coinciding).mp. [mp=title,	275714	Advanced	Display Results | More	

Contract

abstract, original title, name of substance word, subject heading word, floating sub-heading word, keyword heading word, organism supplementary concept word, protocol supplementary concept word, rare disease supplementary concept word, unique identifier, synonyms]

9	4 and 7	350980	Advanced	Display Results | More	
10	8 and 9	23259	Advanced	Display Results | More	
11	9 or 10 [this is mental illness addictions, and dual diagnosis combined]	350980	Advanced	Display Results | More	
12	guideline/ or exp practice guideline/	33535	Advanced	Display Results | More	
13	(Guideline* or consensus or best practice or recommendation*).mp. [mp=title, abstract, original title, name of substance word, subject heading word, floating sub-heading word, keyword heading word, organism supplementary concept word, protocol supplementary concept word, rare disease supplementary concept word, unique identifier, synonyms]	781799	Advanced	Display Results | More	
14	12 or 13	781799	Advanced	Display Results | More	
15	11 and 14	11936	Advanced	Display Results | More	
16	limit 15 to (english language and yr="2000 -Current")	9032	Advanced	Display Results | More	
17	11 and 12	454	Advanced	Display Results | More	

Save Remove Combine with: AND OR

Save All Edit Create RSS View Saved

Basic Search | Find Citation | Search Tools | Search Fields | **Advanced Search** | Multi-Field Search

1 Resource selected | Hide | Change

Ovid MEDLINE(R) and Epub Ahead of Print, In-Process & Other Non-Indexed Citations, Daily and Versions(R) 1946 to March 23, 2020

Enter keyword or phrase (* or $ for truncation) **Keyword** Author Title Journal Search

Limits *(expand)* Include Multimedia Map Term to Subject Heading

To search Open Access content on Ovid, go to Basic Search.

Options

Print Email Export + My Projects Keep Selected

Search Information

You searched:

11 and 12

Search terms used:

alcohol
alcohol-related
disorders
amphetamine-related
anxiety
behaviou?r
bipolar
and
related
cannabis
club
drugs

All Range Clear 100 Per Page 1 Go Next ›

1. **Update of the Mental Health Gap Action Programme (mhGAP) Guidelines for Mental, Neurological and Substance Use Disorders, 2015**

Anonymous.

World Health Organization. WHO Guidelines Approved by the Guidelines Review Committee 2015 05.

[Review. Practice Guideline]

UI: 26937539

Book Title
Update of the Mental Health Gap Action Programme (mhGAP) Guidelines for Mental, Neurological and Substance Use Disorders, 2015

Abstract Reference
Complete Reference

Full Text
UBC eLink

co-existing
co-occurring
cocaine-related
coinciding
comorbidity
concurrent
crystal
meth
depressant*
depression
diagnos#s
disease*
disorder*
disruptive,
impulse
control,
conduct
dissociative
drug
overdose
dual
diagnos*
elimination
feeding
eating
ghp
guideline
hallucinogen*
heroin
illness*
inhalant
abuse
insomnia
lsd
mania
marijuana
mdma
mental
methamphetamine
mood
narcotic-related
neurotic
nicotine
paraphilic
personality
phencyclidine
post
traumatic
stress
practice
prescription
narcotic*
psychiatric
psychoses,
substance-induced
psychosis
schizo*
schizophrenia
spectrum
other
psychotic
sexual
dysfunctions,
psychological
sleep
wake

▶ Abstract + My Projects + Annotate

---

2. **Using Technology-Based Therapeutic Tools in Behavioral Health Services**

Center for Substance Abuse Treatment (US).

Substance Abuse and Mental Health Services Administration (US). SAMHSA/CSAT Treatment Improvement Protocols, Report No.: (SMA) 15-4924. 2015.

[Practice Guideline]

UI: 26889536

Book Title
Using Technology-Based Therapeutic Tools in Behavioral Health Services

Authors Full Name
Center for Substance Abuse Treatment (US).

▶ Abstract + My Projects + Annotate

Abstract Reference
Complete Reference

Full Text
UBC eLink

---

3. **Pregnancy, Childbirth, Postpartum and Newborn Care: A Guide for Essential Practice**

Anonymous.

World Health Organization. 3rd WHO Guidelines Approved by the Guidelines Review Committee 2015.

[Review. Practice Guideline]

UI: 26561684

Book Title
Pregnancy, Childbirth, Postpartum and Newborn Care: A Guide for Essential Practice

▶ Abstract + My Projects + Annotate

Abstract Reference
Complete Reference

Full Text
UBC eLink

---

4. **Trauma-Informed Care in Behavioral Health Services**

Center for Substance Abuse Treatment (US).

Substance Abuse and Mental Health Services Administration (US). SAMHSA/CSAT Treatment Improvement Protocols, Report No.: (SMA) 14-4816. 2014.

[Practice Guideline]

UI: 24901203

Book Title
Trauma-Informed Care in Behavioral Health Services

Authors Full Name
Center for Substance Abuse Treatment (US).

▶ Abstract + My Projects + Annotate

Abstract Reference
Complete Reference

Full Text
UBC eLink

---

5. **WHO Recommendations for the Prevention and Management of Tobacco Use and Second-Hand Smoke Exposure in Pregnancy**

Anonymous.

World Health Organization. WHO Guidelines Approved by the Guidelines Review Committee 2013.

Abstract Reference
Complete Reference

Full Text
UBC eLink

somatoform
stimulant
stimulant*
substance
abuse,
intravenous
oral
withdrawal
syndrome
substance-related
tobacco
use
disorder
trauma
stressor

Search Returned:
454 text results

Sort By:

-

Customize Display

Filter By

Add to Search History

Selected Only (0)

- **Years**
  - **All Years**
  - Current year
  - Past 3 years
  - Past 5 years
  - **Specific Year Range**
- **Subject**
- **Author**
- **Journal**
- **Book**
- **Publication Type**

My Projects

+ New Project

No projects available.

JBI EBP Tools

SUMARI

[Review. Practice Guideline]

UI: 24649520

Book Title
WHO Recommendations for the Prevention and Management of Tobacco Use and Second-Hand Smoke Exposure in Pregnancy

Abstract + My Projects + Annotate

---

6. **Self-Harm: Longer-Term Management**

National Collaborating Centre for Mental Health (UK).

British Psychological Society. National Institute for Health and Clinical Excellence: Guidance 2012.

[Review. Practice Guideline]

UI: 23534084

Book Title
Self-Harm: Longer-Term Management

Authors Full Name
National Collaborating Centre for Mental Health (UK).

Abstract + My Projects + Annotate

Abstract Reference
Complete Reference

Full Text
UBC eLink

---

7. **Generalised Anxiety Disorder in Adults: Management in Primary, Secondary and Community Care**

National Collaborating Centre for Mental Health (UK).

British Psychological Society. National Institute for Health and Clinical Excellence: Guidance 2011.

[Review. Practice Guideline]

UI: 22536620

Book Title
Generalised Anxiety Disorder in Adults: Management in Primary, Secondary and Community Care

Authors Full Name
National Collaborating Centre for Mental Health (UK).

Abstract + My Projects + Annotate

Abstract Reference
Complete Reference

Full Text
UBC eLink

---

8. **Psychosis with Coexisting Substance Misuse: Assessment and Management in Adults and Young People**

National Collaborating Centre for Mental Health (UK).

British Psychological Society. National Institute for Health and Clinical Excellence: Guidance 2011.

[Review. Practice Guideline]

UI: 23115814

Book Title
Psychosis with Coexisting Substance Misuse: Assessment and Management in Adults and Young People

Authors Full Name
National Collaborating Centre for Mental Health (UK).

Abstract + My Projects + Annotate

Abstract Reference
Complete Reference

Full Text
UBC eLink

9. **mhGAP Intervention Guide for Mental, Neurological and Substance Use Disorders in Non-Specialized Health Settings: Mental Health Gap Action Programme (mhGAP)**

Abstract Reference
Complete Reference

Anonymous.

World Health Organization. WHO Guidelines Approved by the Guidelines Review Committee 2010.

Full Text
UBC eLink

[Review. Practice Guideline]

UI: 23741783

Book Title
mhGAP Intervention Guide for Mental, Neurological and Substance Use Disorders in Non-Specialized Health Settings: Mental Health Gap Action Programme (mhGAP)

▶ Abstract + My Projects + Annotate

---

10. **Pharmacological Treatment of Mental Disorders in Primary Health Care**

Abstract Reference
Complete Reference

Anonymous.

World Health Organization. WHO Guidelines Approved by the Guidelines Review Committee 2009.

Full Text
UBC eLink

[Review. Practice Guideline]

UI: 23762966

Book Title
Pharmacological Treatment of Mental Disorders in Primary Health Care

▶ Abstract + My Projects + Annotate

---

11. **Managing Depressive Symptoms in Substance Abuse Clients During Early Recovery**

Abstract Reference
Complete Reference

Center for Substance Abuse Treatment.

Substance Abuse and Mental Health Services Administration (US). SAMHSA/CSAT Treatment Improvement Protocols, Report No.: (SMA)08-4353. 2008.

Full Text
UBC eLink

[Practice Guideline]

UI: 22514854

Book Title
Managing Depressive Symptoms in Substance Abuse Clients During Early Recovery

Authors Full Name
Center for Substance Abuse Treatment.

▶ Abstract + My Projects + Annotate

---

12. **mhGAP: Mental Health Gap Action Programme: Scaling Up Care for Mental, Neurological and Substance Use Disorders**

Abstract Reference
Complete Reference

Anonymous.

World Health Organization. WHO Guidelines Approved by the Guidelines Review Committee 2008.

Full Text
UBC eLink

[Review. Practice Guideline]

UI: 26290926

Book Title
mhGAP: Mental Health Gap Action Programme: Scaling Up Care for Mental, Neurological and Substance Use Disorders

Abstract + My Projects + Annotate

---

13. **Substance Abuse Treatment for Persons With Co-Occurring Disorders**

Abstract Reference
Complete Reference

Full Text
UBC eLink

Center for Substance Abuse Treatment.

Substance Abuse and Mental Health Services Administration (US). SAMHSA/CSAT Treatment Improvement Protocols, Report No.: (SMA) 05-3922. 2005.

[Practice Guideline]

UI: 22514848

Book Title
Substance Abuse Treatment for Persons With Co-Occurring Disorders

Authors Full Name
Center for Substance Abuse Treatment.

Abstract + My Projects + Annotate

---

14. **Substance Abuse Treatment for Persons with HIV/AIDS**

Abstract Reference
Complete Reference

Full Text
UBC eLink

Center for Substance Abuse Treatment.

Substance Abuse and Mental Health Services Administration (US). SAMHSA/CSAT Treatment Improvement Protocols, Report No.: (SMA) 00-3410. 2000.

[Practice Guideline]

UI: 22514843

Book Title
Substance Abuse Treatment for Persons with HIV/AIDS

Authors Full Name
Center for Substance Abuse Treatment.

Abstract + My Projects + Annotate

---

15. **Continuity of Offender Treatment for Substance Use Disorders from Institution to Community**

Abstract Reference
Complete Reference

Full Text
UBC eLink

Center for Substance Abuse Treatment.

Substance Abuse and Mental Health Services Administration (US). SAMHSA/CSAT Treatment Improvement Protocols, Report No.: (SMA) 98-3245. 1998.

[Practice Guideline]

UI: 22514836

Book Title
Continuity of Offender Treatment for Substance Use Disorders from Institution to Community

Authors Full Name
Center for Substance Abuse Treatment.

Abstract + My Projects + Annotate

16. **Assessment and Treatment of Patients with Coexisting Mental Illness and Alcohol and Other Drug Abuse**

Center for Substance Abuse Treatment.

Substance Abuse and Mental Health Services Administration (US). SAMHSA/CSAT Treatment Improvement Protocols, Report No.: (SMA) 95-3061. 1994.

[Practice Guideline]

UI: 22514866

Book Title
Assessment and Treatment of Patients with Coexisting Mental Illness and Alcohol and Other Drug Abuse

Authors Full Name
Center for Substance Abuse Treatment.

Abstract + My Projects + Annotate

Abstract Reference
Complete Reference

Full Text
UBC eLink

17. **Metabolic risk reduction in patients with schizophrenia treated with antipsychotics: recommendations of the Polish Psychiatric Association.** Redukcja ryzyka metabolicznego u chorych na schizofrenie przyjmujacych leki przeciwpsychotyczne - zalecenia Polskiego Towarzystwa Psychiatrycznego.

Wichniak A; Dudek D; Heitzman J; Kaplon-Cieslicka A; Mamcarz A; Samochowiec J; Szulc A; Bienkowski P.

Psychiatria Polska. 53(6):1191-1218, 2019 Dec 31.

[Guideline]

UI: 32017812

Authors Full Name
Wichniak, Adam; Dudek, Dominika; Heitzman, Janusz; Kaplon-Cieslicka, Agnieszka; Mamcarz, Artur; Samochowiec, Jerzy; Szulc, Agata; Bienkowski, Przemyslaw.

Abstract + My Projects + Annotate

Abstract Reference
Complete Reference

Find Similar
Find Citing Articles

Bibliographic Links
UBC eLink

18. **Guidelines of the French Society of Otorhinolaryngology. Role of the ENT specialist in the diagnosis of obstructive sleep apnea-hypopnea syndrome (OSAHS) in children. Part 2: Diagnostic investigations apart from sleep studies.**

Leclere JC; Marianowski R; Monteyrol PJ; Akkari M; Chalumeau F; Fayoux P; Leboulanger N; Franco P; Couloigner V; Mondain M.

European annals of otorhinolaryngology, head & neck diseases. 136(4):295-299, 2019 Sep.

[Journal Article. Practice Guideline]

UI: 31202665

Authors Full Name
Leclere, J-C; Marianowski, R; Monteyrol, P J; Akkari, M; Chalumeau, F; Fayoux, P; Leboulanger, N; Franco, P; Couloigner, V; Mondain, M.

Abstract + My Projects + Annotate

Abstract Reference
Complete Reference

Find Similar
Find Citing Articles

Bibliographic Links
UBC eLink

19. **Recommendations for the treatment of schizophrenia with negative**

symptoms. Standards of pharmacotherapy by the Polish Psychiatric Association (Polskie Towarzystwo Psychiatryczne), part 2.
Rekomendacje dotyczace leczenia schizofrenii z objawami negatywnymi. Standardy farmakoterapii Polskiego Towarzystwa Psychiatrycznego, czesc 2.

Szulc A; Dudek D; Samochowiec J; Wojnar M; Heitzman J; Galecki P.

Psychiatria Polska. 53(3):525-540, 2019 Jun 30.

[Guideline]

UI: 31522194

Authors Full Name
Szulc, Agata; Dudek, Dominika; Samochowiec, Jerzy; Wojnar, Marcin; Heitzman, Janusz; Galecki, Piotr.

Abstract + My Projects + Annotate

Abstract Reference
Complete Reference

Find Similar
Find Citing Articles

Bibliographic Links
UBC eLink

---

20. **Recommendations for the treatment of schizophrenia with negative symptoms. Standards of pharmacotherapy by the Polish Psychiatric Association (Polskie Towarzystwo Psychiatryczne), part 1. [Review]**
Rekomendacje dotyczace leczenia schizofrenii z objawami negatywnymi. Standardy farmakoterapii Polskiego Towarzystwa Psychiatrycznego, czesc 1.

Szulc A; Samochowiec J; Galecki P; Wojnar M; Heitzman J; Dudek D.

Psychiatria Polska. 53(3):497-524, 2019 Jun 30.

[Guideline. Journal Article. Review]

UI: 31522193

Authors Full Name
Szulc, Agata; Samochowiec, Jerzy; Galecki, Piotr; Wojnar, Marcin; Heitzman, Janusz; Dudek, Dominika.

Abstract + My Projects + Annotate

Abstract Reference
Complete Reference

Find Similar
Find Citing Articles

Bibliographic Links
UBC eLink

---

21. **Mental Health Competencies for Pediatric Practice.**

Foy JM; Green CM; Earls MF; COMMITTEE ON PSYCHOSOCIAL ASPECTS OF CHILD AND FAMILY HEALTH, MENTAL HEALTH LEADERSHIP WORK GROUP.

Pediatrics. 144(5), 2019 11.

[Journal Article. Practice Guideline]

UI: 31636143

Authors Full Name
Foy, Jane Meschan; Green, Cori M; Earls, Marian F; COMMITTEE ON PSYCHOSOCIAL ASPECTS OF CHILD AND FAMILY HEALTH, MENTAL HEALTH LEADERSHIP WORK GROUP.

Abstract + My Projects + Annotate

Abstract Reference
Complete Reference

Find Similar
Find Citing Articles

Bibliographic Links
UBC eLink

---

22. **American Society for Enhanced Recovery and Perioperative Quality Initiative-4 Joint Consensus Statement on Persistent Postoperative Opioid Use: Definition, Incidence, Risk Factors, and Health Care System Initiatives.**

Kent ML; Hurley RW; Oderda GM; Gordon DB; Sun E; Mythen M; Miller TE; Shaw AD; Gan TJ; Thacker JKM; McEvoy MD; POQI-4 Working Group.

Anesthesia & Analgesia. 129(2):543-552, 2019 08.

Ovid Full Text
Abstract Reference
Complete Reference

Find Similar
Find Citing Articles

[Journal Article. Practice Guideline. Research Support, Non-U.S. Gov't. Systematic Review]

UI: 30897590

Title Comment

[Comment in: Anesth Analg. 2019 Aug;129(2):324-326; PMID: 31313668 [https://www-ncbi-nlm-nih-gov.ezproxy.library.ubc.ca/pub...]]

Authors Full Name

Kent, Michael L; Hurley, Robert W; Oderda, Gary M; Gordon, Debra B; Sun, Eric; Mythen, Monty; Miller, Timothy E; Shaw, Andrew D; Gan, Tong J; Thacker, Julie K M; McEvoy, Matthew D; POQI-4 Working Group.

Abstract | Article as PDF (620KB) | + My Projects | + Annotate

Full Text
Bibliographic Links
UBC eLink

---

23. **Society for Obstetric Anesthesia and Perinatology Consensus Statement: Monitoring Recommendations for Prevention and Detection of Respiratory Depression Associated With Administration of Neuraxial Morphine for Cesarean Delivery Analgesia.**

Bauchat JR; Weiniger CF; Sultan P; Habib AS; Ando K; Kowalczyk JJ; Kato R; George RB; Palmer CM; Carvalho B.

Anesthesia & Analgesia. 129(2):458-474, 2019 08.

[Journal Article. Practice Guideline. Systematic Review]

UI: 31082964

Title Comment

[Comment in: Anesth Analg. 2019 Aug;129(2):330-332; PMID: 31313670 [https://www-ncbi-nlm-nih-gov.ezproxy.library.ubc.ca/pub...]]

Authors Full Name

Bauchat, Jeanette R; Weiniger, Carolyn F; Sultan, Pervez; Habib, Ashraf S; Ando, Kazuo; Kowalczyk, John J; Kato, Rie; George, Ronald B; Palmer, Craig M; Carvalho, Brendan.

Abstract | Article as PDF (659KB) | + My Projects | + Annotate

Ovid Full Text
Abstract Reference
Complete Reference

Find Similar
Find Citing Articles

Bibliographic Links
UBC eLink

---

24. **Clinical Practice Guideline for the Diagnosis, Evaluation, and Treatment of Attention-Deficit/Hyperactivity Disorder in Children and Adolescents.**

Wolraich ML; Hagan JF Jr; Allan C; Chan E; Davison D; Earls M; Evans SW; Flinn SK; Froehlich T; Frost J; Holbrook JR; Lehmann CU; Lessin HR; Okechukwu K; Pierce KL; Winner JD; Zurhellen W; SUBCOMMITTEE ON CHILDREN AND ADOLESCENTS WITH ATTENTION-DEFICIT/HYPERACTIVE DISORDER.

Pediatrics. 144(4), 2019 10.

[Journal Article. Practice Guideline]

UI: 31570648

Authors Full Name

Wolraich, Mark L; Hagan, Joseph F Jr; Allan, Carla; Chan, Eugenia; Davison, Dale; Earls, Marian; Evans, Steven W; Flinn, Susan K; Froehlich, Tanya; Frost, Jennifer; Holbrook, Joseph R; Lehmann, Christoph Ulrich; Lessin, Herschel Robert; Okechukwu, Kymika; Pierce, Karen L; Winner, Jonathan D; Zurhellen, William; SUBCOMMITTEE ON CHILDREN AND ADOLESCENTS WITH ATTENTION-DEFICIT/HYPERACTIVE DISORDER.

Abstract | + My Projects | + Annotate

Abstract Reference
Complete Reference

Find Similar
Find Citing Articles

Bibliographic Links
UBC eLink

25. **Recommendations of the Polish Psychiatric Association regarding the treatment of affective disorders in women of childbearing age. Part II: Bipolar disorder.** Rekomendacje Polskiego Towarzystwa Psychiatrycznego dotyczace leczenia zaburzen afektywnych u kobiet w wieku rozrodczym. Czesc II: Choroba afektywna dwubiegunowa.

Rybakowski J; Cubala WJ; Galecki P; Rymaszewska J; Samochowiec J; Szulc A; Dudek D.

Psychiatria Polska. 53(2):263-276, 2019 Apr 30.

[Guideline]

UI: 31317957

Authors Full Name
Rybakowski, Janusz; Cubala, Wieslaw Jerzy; Galecki, Piotr; Rymaszewska, Joanna; Samochowiec, Jerzy; Szulc, Agata; Dudek, Dominika.

Abstract + My Projects + Annotate

Abstract Reference
Complete Reference
Find Similar
Find Citing Articles
Bibliographic Links
UBC eLink

---

26. **Recommendations of the Polish Psychiatric Association for treatment of affective disorders in women of childbearing age. Part I: Treatment of depression.** Rekomendacje Polskiego Towarzystwa Psychiatrycznego dotyczace leczenia zaburzen afektywnych u kobiet w wieku rozrodczym. Czesc I: Leczenie depresji.

Samochowiec J; Rybakowski J; Galecki P; Szulc A; Rymaszewska J; Cubala WJ; Dudek D.

Psychiatria Polska. 53(2):245-262, 2019 04 30.

[Guideline]

UI: 31317956

Authors Full Name
Samochowiec, Jerzy; Rybakowski, Janusz; Galecki, Piotr; Szulc, Agata; Rymaszewska, Joanna; Cubala, Wieslaw Jerzy; Dudek, Dominika.

Abstract + My Projects + Annotate

Abstract Reference
Complete Reference
Find Similar
Find Citing Articles
Bibliographic Links
UBC eLink

---

27. **Pharmacological treatment of obsessive compulsive disorder in adults: A clinical practice guideline based on the ADAPTE methodology.** Tratamiento farmacologico del trastorno obsesivo-compulsivo en adultos: una guia de practica clinica basada en el metodo ADAPTE.

Menchon JM; Bobes J; Alamo C; Alonso P; Garcia-Portilla MP; Ibanez A; Real E; Bousono M; Saiz-Gonzalez MD; Saiz-Ruiz J.

Revista de Psiquiatria y Salud Mental. 12(2):77-91, 2019 Apr - Jun.

[Consensus Development Conference. Journal Article. Practice Guideline]

UI: 30850318

Authors Full Name
Menchon, Jose M; Bobes, Julio; Alamo, Cecilio; Alonso, Pino; Garcia-Portilla, Maria Paz; Ibanez, Angela; Real, Eva; Bousono, Manuel; Saiz-Gonzalez, Maria Dolores; Saiz-Ruiz, Jeronimo.

Abstract + My Projects + Annotate

Abstract Reference
Complete Reference
Find Similar
Find Citing Articles
Bibliographic Links
UBC eLink

---

28. **ASIPP Guidelines for Sedation and Fasting Status of Patients Undergoing Interventional Pain Management Procedures.**

Abstract Reference
Complete Reference

Kaye AD; Jones MR; Viswanath O; Candido KD; Boswell MV; Soin A; Sanapati M; Harned ME; Simopoulos TT; Diwan S; Albers SL; Datta S; Falco FJ; Manchikanti L.

Pain Physician. 22(3):201-207, 2019 05.

[Journal Article. Practice Guideline]

UI: 31151329

Authors Full Name
Kaye, Alan D; Jones, Mark R; Viswanath, Omar; Candido, Kenneth D; Boswell, Mark V; Soin, Amol; Sanapati, Mahendra; Harned, Michael E; Simopoulos, Thomas T; Diwan, Sudhir; Albers, Sheri L; Datta, Sukdeb; Falco, Frank Je; Manchikanti, Laxmaiah.

Abstract + My Projects + Annotate

Find Similar
Find Citing Articles

Bibliographic Links
UBC eLink

---

29. **Italian Association of Sleep Medicine (AIMS) position statement and guideline on the treatment of menopausal sleep disorders.**

Silvestri R; Arico I; Bonanni E; Bonsignore M; Caretto M; Caruso D; Di Perri MC; Galletta S; Lecca RM; Lombardi C; Maestri M; Miccoli M; Palagini L; Provini F; Puligheddu M; Savarese M; Spaggiari MC; Simoncini T.

Maturitas. 129:30-39, 2019 Nov.

[Journal Article. Practice Guideline. Systematic Review]

UI: 31547910

Authors Full Name
Silvestri, R; Arico, I; Bonanni, E; Bonsignore, M; Caretto, M; Caruso, D; Di Perri, M C; Galletta, S; Lecca, R M; Lombardi, C; Maestri, M; Miccoli, M; Palagini, L; Provini, F; Puligheddu, M; Savarese, M; Spaggiari, M C; Simoncini, T.

Abstract + My Projects + Annotate

Abstract Reference
Complete Reference

Find Similar
Find Citing Articles

Bibliographic Links
UBC eLink

---

30. **Joint BAP NAPICU evidence-based consensus guidelines for the clinical management of acute disturbance: De-escalation and rapid tranquillisation.**

Patel MX; Sethi FN; Barnes TR; Dix R; Dratcu L; Fox B; Garriga M; Haste JC; Kahl KG; Lingford-Hughes A; McAllister-Williams H; O'Brien A; Parker C; Paterson B; Paton C; Posporelis S; Taylor DM; Vieta E; Vollm B; Wilson-Jones C; Woods L; With co-authors (in alphabetical order):.

Journal of Psychopharmacology. 32(6):601-640, 2018 06.

[Journal Article. Practice Guideline. Research Support, Non-U.S. Gov't]

UI: 29882463

Authors Full Name
Patel, Maxine X; Sethi, Faisil N; Barnes, Thomas Re; Dix, Roland; Dratcu, Luiz; Fox, Bernard; Garriga, Marina; Haste, Julie C; Kahl, Kai G; Lingford-Hughes, Anne; McAllister-Williams, Hamish; O'Brien, Aileen; Parker, Caroline; Paterson, Brodie; Paton, Carol; Posporelis, Sotiris; Taylor, David M; Vieta, Eduard; Vollm, Birgit; Wilson-Jones, Charlotte; Woods, Laura; With co-authors (in alphabetical order):.

Abstract + My Projects + Annotate

Abstract Reference
Complete Reference

Find Similar
Find Citing Articles

Bibliographic Links
UBC eLink

---

31. **Thyroid hormones treatment for subclinical hypothyroidism: a clinical practice guideline.**

Bekkering GE; Agoritsas T; Lytvyn L; Heen AF; Feller M; Moutzouri E; Abdulazeem H; Aertgeerts B; Beecher D; Brito JP; Farhoumand PD; Singh

Abstract Reference
Complete Reference

Ospina N; Rodondi N; van Driel M; Wallace E; Snel M; Okwen PM; Siemieniuk R; Vandvik PO; Kuijpers T; Vermandere M.

BMJ. 365:l2006, 2019 May 14.

[Journal Article. Practice Guideline]

UI: 31088853

Title Comment

[Comment in: Nat Rev Endocrinol. 2019 Sep;15(9):503-504; PMID: 31300726 [https://www-ncbi-nlm-nih-gov.ezproxy.library.ubc.ca/pub...]]

Authors Full Name

Bekkering, G E; Agoritsas, T; Lytvyn, L; Heen, A F; Feller, M; Moutzouri, E; Abdulazeem, H; Aertgeerts, B; Beecher, D; Brito, J P; Farhoumand, P D; Singh Ospina, N; Rodondi, N; van Driel, M; Wallace, E; Snel, M; Okwen, P M; Siemieniuk, R; Vandvik, P O; Kuijpers, T; Vermandere, M.

▶ Abstract + My Projects + Annotate

Find Similar
Find Citing Articles

Bibliographic Links
UBC eLink

---

32. **EPA guidance on tobacco dependence and strategies for smoking cessation in people with mental illness.**

Ruther T; Bobes J; De Hert M; Svensson TH; Mann K; Batra A; Gorwood P; Moller HJ; European Psychiatric Association.

European Psychiatry: the Journal of the Association of European Psychiatrists. 29(2):65-82, 2014 Feb.

[Journal Article. Practice Guideline. Research Support, Non-U.S. Gov't]

UI: 24485753

Authors Full Name

Ruther, T; Bobes, J; De Hert, M; Svensson, T H; Mann, K; Batra, A; Gorwood, P; Moller, H J; European Psychiatric Association.

▶ Abstract + My Projects + Annotate

Abstract Reference
Complete Reference

Find Similar
Find Citing Articles

Bibliographic Links
UBC eLink

---

33. **Position statement of the European Psychiatric Association (EPA) on the value of antidepressants in the treatment of unipolar depression.**

Moller HJ; Bitter I; Bobes J; Fountoulakis K; Hoschl C; Kasper S; European Psychiatric Association.

European Psychiatry: the Journal of the Association of European Psychiatrists. 27(2):114-28, 2012 Feb.

[Journal Article. Practice Guideline. Research Support, Non-U.S. Gov't]

UI: 22119161

Authors Full Name

Moller, H-J; Bitter, I; Bobes, J; Fountoulakis, K; Hoschl, C; Kasper, S; European Psychiatric Association.

▶ Abstract + My Projects + Annotate

Abstract Reference
Complete Reference

Find Similar
Find Citing Articles

Bibliographic Links
UBC eLink

---

34. **The European Psychiatric Association (EPA) guidance on suicide treatment and prevention.**

Wasserman D; Rihmer Z; Rujescu D; Sarchiapone M; Sokolowski M; Titelman D; Zalsman G; Zemishlany Z; Carli V; European Psychiatric Association.

European Psychiatry: the Journal of the Association of European Psychiatrists. 27(2):129-41, 2012 Feb.

[Journal Article. Practice Guideline]

UI: 22137775

Abstract Reference
Complete Reference

Find Similar
Find Citing Articles

Bibliographic Links

Authors Full Name
Wasserman, D; Rihmer, Z; Rujescu, D; Sarchiapone, M; Sokolowski, M; Titelman, D; Zalsman, G; Zemishlany, Z; Carli, V; European Psychiatric Association.

UBC eLink

▶ Abstract + My Projects + Annotate

---

35. **Second joint position paper: Use of isotretinoin in severe acne. [Spanish]** Segunda declaracion de posicion conjunta: uso de isotretinoina en acne severo

Gomez-Flores M; Poletti-Vazquez DE; Garcia-Hidalgo L; Fierro-Arias L; Herz-Ruelas M; Garza-Gomez J; Rosas-Ortiz JF.

Revista Medica del Instituto Mexicano del Seguro Social. 56(5):441-446, 2019 Jan 28.

[Consensus Development Conference. Journal Article. Practice Guideline]

UI: 30777411

Authors Full Name
Gomez-Flores, Minerva; Poletti-Vazquez, David Eduardo; Garcia-Hidalgo, Linda; Fierro-Arias, Leonel; Herz-Ruelas, Maira; Garza-Gomez, Jorge; Rosas-Ortiz, Jose Federico.

Abstract Reference
Complete Reference
Find Similar
Find Citing Articles
Bibliographic Links
UBC eLink

▶ Abstract + My Projects + Annotate

---

36. **Joint AAD-NPF guidelines of care for the management and treatment of psoriasis with awareness and attention to comorbidities. [Review]**

Elmets CA; Leonardi CL; Davis DMR; Gelfand JM; Lichten J; Mehta NN; Armstrong AW; Connor C; Cordoro KM; Elewski BE; Gordon KB; Gottlieb AB; Kaplan DH; Kavanaugh A; Kivelevitch D; Kiselica M; Korman NJ; Kroshinsky D; Lebwohl M; Lim HW; Paller AS; Parra SL; Pathy AL; Prater EF; Rupani R; Siegel M; Stoff B; Strober BE; Wong EB; Wu JJ; Hariharan V; Menter A.

Journal of the American Academy of Dermatology. 80(4):1073-1113, 2019 Apr.

[Journal Article. Practice Guideline. Review]

UI: 30772097

Authors Full Name
Elmets, Craig A; Leonardi, Craig L; Davis, Dawn M R; Gelfand, Joel M; Lichten, Jason; Mehta, Nehal N; Armstrong, April W; Connor, Cody; Cordoro, Kelly M; Elewski, Boni E; Gordon, Kenneth B; Gottlieb, Alice B; Kaplan, Daniel H; Kavanaugh, Arthur; Kivelevitch, Dario; Kiselica, Matthew; Korman, Neil J; Kroshinsky, Daniela; Lebwohl, Mark; Lim, Henry W; Paller, Amy S; Parra, Sylvia L; Pathy, Arun L; Prater, Elizabeth Farley; Rupani, Reena; Siegel, Michael; Stoff, Benjamin; Strober, Bruce E; Wong, Emily B; Wu, Jashin J; Hariharan, Vidhya; Menter, Alan.

Abstract Reference
Complete Reference
Find Similar
Find Citing Articles
Bibliographic Links
UBC eLink

▶ Abstract + My Projects + Annotate

---

37. **ACOG Committee Opinion No. 762: Prepregnancy Counseling.**

Anonymous.

Obstetrics & Gynecology. 133(1):e78-e89, 2019 01.

[Journal Article. Practice Guideline]

UI: 30575679

Ovid Full Text
Abstract Reference
Complete Reference
Find Similar
Find Citing Articles

▶ Abstract Article as PDF (220KB) + My Projects + Annotate

Bibliographic Links

UBC eLink

38. **ACOG Committee Opinion No. 762 Summary: Prepregnancy Counseling.**

Anonymous.

Obstetrics & Gynecology. 133(1):228-230, 2019 01.

[Journal Article. Practice Guideline]

UI: 30575672

Abstract | Article as PDF (123KB) | + My Projects | + Annotate

Ovid Full Text
Abstract Reference
Complete Reference

Find Similar
Find Citing Articles

Bibliographic Links
UBC eLink

39. **[The 10-year findings from the FondaMental Academic Center of Expertise for Schizophrenia (FACE-SZ): Review and recommendations for clinical practice]. [French]** Centres Experts Schizophrenie, un outil pour le soin et la recherche : retour sur 10 ans d'experience.

Schurhoff F; Fond G; Berna F; Bulzacka E; Godin O; Boyer L; Misdrahi D; Andrianarisoa M; Brunel L; Coulon N; Aouizerate B; Capdevielle D; Chereau I; D'Amato T; Dubertret C; Dubreucq J; Faget C; Gabayet F; Mallet J; Rey R; Lancon C; Passerieux C; Schandrin A; Urbach M; Vidailhet P; Leboyer M; Llorca PM; FACE-SZ (FondaMental Academic Centers of Expertise for Schizophrenia) group.

Encephale. 45(1):9-14, 2019 Feb.

[Guideline. Journal Article]

UI: 30327207

Title Comment
[Comment in: Encephale. 2019 Feb;45(1):90-91; PMID: 29784520 [https://www-ncbi-nlm-nih-gov.ezproxy.library.ubc.ca/pub...]]

Authors Full Name
Schurhoff, F; Fond, G; Berna, F; Bulzacka, E; Godin, O; Boyer, L; Misdrahi, D; Andrianarisoa, M; Brunel, L; Coulon, N; Aouizerate, B; Capdevielle, D; Chereau, I; D'Amato, T; Dubertret, C; Dubreucq, J; Faget, C; Gabayet, F; Mallet, J; Rey, R; Lancon, C; Passerieux, C; Schandrin, A; Urbach, M; Vidailhet, P; Leboyer, M; Llorca, P M; FACE-SZ (FondaMental Academic Centers of Expertise for Schizophrenia) group.

Abstract | + My Projects | + Annotate

Abstract Reference
Complete Reference

Find Similar
Find Citing Articles

Bibliographic Links
UBC eLink

40. **[Clinical guidelines for the management of schizophrenia: Pharmacological and psychological interventions (III)]. [Greek]**

Hadjulis M; Margariti M; Lazaridou M; Angelidis GF; Fotopoulos V; Markaki L; Koulouri F.

Psychiatriki. 29(4):303-315, 2018 Oct-Dec.

[Guideline. Journal Article]

UI: 30814040

Authors Full Name
Hadjulis, M; Margariti, M; Lazaridou, M; Angelidis, G F; Fotopoulos, V; Markaki, L; Koulouri, F.

Abstract | + My Projects | + Annotate

Abstract Reference
Complete Reference

Find Similar
Find Citing Articles

Bibliographic Links
UBC eLink

41. **Screening for Intimate Partner Violence, Elder Abuse, and Abuse of Vulnerable Adults: US Preventive Services Task Force Final Recommendation Statement.**

US Preventive Services Task Force; Curry SJ; Krist AH; Owens DK; Barry MJ; Caughey AB; Davidson KW; Doubeni CA; Epling JW Jr; Grossman DC; Kemper AR; Kubik M; Kurth A; Landefeld CS; Mangione CM; Silverstein M; Simon MA; Tseng CW; Wong JB.

JAMA. 320(16):1678-1687, 2018 10 23.

[Journal Article. Practice Guideline. Research Support, U.S. Gov't, P.H.S.]

UI: 30357305

Title Comment
[Comment in: JAMA. 2018 Oct 23;320(16):1645-1647; PMID: 30357278 [https://www-ncbi-nlm-nih-gov.ezproxy.library.ubc.ca/pub...]]
[Summary for Patients in: JAMA. 2018 Oct 23;320(16):1718; PMID: 30357300 [https://www-ncbi-nlm-nih-gov.ezproxy.library.ubc.ca/pub...]]

Authors Full Name
US Preventive Services Task Force; Curry, Susan J; Krist, Alex H; Owens, Douglas K; Barry, Michael J; Caughey, Aaron B; Davidson, Karina W; Doubeni, Chyke A; Epling, John W Jr; Grossman, David C; Kemper, Alex R; Kubik, Martha; Kurth, Ann; Landefeld, C Seth; Mangione, Carol M; Silverstein, Michael; Simon, Melissa A; Tseng, Chien-Wen; Wong, John B.

▶ Abstract + My Projects + Annotate

Abstract Reference
Complete Reference
Find Similar
Find Citing Articles
Bibliographic Links
UBC eLink

---

42. **French Society of Otorhinolaryngology and Head and Neck Surgery (SFORL) guidelines concerning the role of otorhinolaryngologists in the management of paediatric obstructive sleep apnoea syndrome: Follow-up protocol for treated children.**

Akkari M; Marianowski R; Chalumeau F; Fayoux P; Leboulanger N; Monteyrol PJ; Mondain M; Groupe de Travail de la SFORL.

European annals of otorhinolaryngology, head & neck diseases. 135(6):427-431, 2018 Dec.

[Journal Article. Practice Guideline]

UI: 30318322

Authors Full Name
Akkari, M; Marianowski, R; Chalumeau, F; Fayoux, P; Leboulanger, N; Monteyrol, P J; Mondain, M; Groupe de Travail de la SFORL.

▶ Abstract + My Projects + Annotate

Abstract Reference
Complete Reference
Find Similar
Find Citing Articles
Bibliographic Links
UBC eLink

---

43. **Academy of Nutrition and Dietetics: Revised 2018 Standards of Practice and Standards of Professional Performance for Registered Dietitian Nutritionists (Competent, Proficient, and Expert) in Mental Health and Addictions.**

Anderson Girard T; Russell K; Leyse-Wallace R.

Journal of the Academy of Nutrition & Dietetics. 118(10):1975-1986.e53, 2018 10.

[Journal Article. Practice Guideline]

UI: 30262106

Authors Full Name
Anderson Girard, Terry; Russell, Kathryn; Leyse-Wallace, Ruth.

Abstract Reference
Complete Reference
Find Similar
Find Citing Articles
Bibliographic Links
UBC eLink

▶ Abstract + My Projects + Annotate

44. **Management of Prader-Willi Syndrome (PWS) in adults - what an endocrinologist needs to know. Recommendations of the Polish Society of Endocrinology and the Polish Society of Paediatric Endocrinology and Diabetology.**

Abstract Reference
Complete Reference

Find Similar
Find Citing Articles

UBC eLink

Goralska M; Bednarczuk T; Roslon M; Libura M; Szalecki M; Hilczer M; Stawerska R; Smyczynska J; Karbownik-Lewinska M; Walczak M; Lewinski A.

Endokrynologia Polska. 69(4), 2018.

[Journal Article. Practice Guideline]

UI: 30209801

Authors Full Name
Goralska, Magdalena; Bednarczuk, Tomasz; Roslon, Marek; Libura, Maria; Szalecki, Mieczyslaw; Hilczer, Maciej; Stawerska, Renata; Smyczynska, Joanna; Karbownik-Lewinska, Malgorzata; Walczak, Mieczyslaw; Lewinski, Andrzej.

▶ Abstract + My Projects + Annotate

45. **Guidelines for the evaluation and treatment of perimenopausal depression: summary and recommendations.**

Ovid Full Text
Abstract Reference
Complete Reference

Find Similar
Find Citing Articles

Bibliographic Links
UBC eLink

Maki PM; Kornstein SG; Joffe H; Bromberger JT; Freeman EW; Athappilly G; Bobo WV; Rubin LH; Koleva HK; Cohen LS; Soares CN; Board of Trustees for The North American Menopause Society (NAMS) and the Women and Mood Disorders Task Force of the National Network of Depression Centers.

Menopause. 25(10):1069-1085, 2018 10.

[Journal Article. Practice Guideline]

UI: 30179986

Authors Full Name
Maki, Pauline M; Kornstein, Susan G; Joffe, Hadine; Bromberger, Joyce T; Freeman, Ellen W; Athappilly, Geena; Bobo, William V; Rubin, Leah H; Koleva, Hristina K; Cohen, Lee S; Soares, Claudio N; Board of Trustees for The North American Menopause Society (NAMS) and the Women and Mood Disorders Task Force of the National Network of Depression Centers.

▶ Abstract Article as PDF (391KB) + My Projects
+ Annotate

46. **First Latin American clinical practice guidelines for the treatment of systemic lupus erythematosus: Latin American Group for the Study of Lupus (GLADEL, *Grupo Latino Americano de Estudio del Lupus*)-Pan-American League of Associations of Rheumatology (PANLAR).**

Abstract Reference
Complete Reference

Find Similar
Find Citing Articles

Full Text
Bibliographic Links
UBC eLink

Pons-Estel BA; Bonfa E; Soriano ER; Cardiel MH; Izcovich A; Popoff F; Criniti JM; Vasquez G; Massardo L; Duarte M; Barile-Fabris LA; Garcia MA; Amigo MC; Espada G; Catoggio LJ; Sato EI; Levy RA; Acevedo Vasquez EM; Chacon-Diaz R; Galarza-Maldonado CM; Iglesias Gamarra AJ; Molina JF; Neira O; Silva CA; Vargas Pena A; Gomez-Puerta JA; Scolnik M; Pons-Estel GJ; Ugolini-Lopes MR; Savio V; Drenkard C; Alvarellos AJ; Ugarte-Gil MF; Babini A; Cavalcanti A; Cardoso Linhares FA; Haye Salinas MJ; Fuentes-Silva YJ; Montandon de Oliveira E Silva AC; Eraso Garnica RM; Herrera Uribe S; Gomez-Martin D; Robaina Sevrini R; Quintana RM; Gordon S; Fragoso-Loyo H; Rosario V; Saurit V; Appenzeller S; Dos Reis Neto ET; Cieza J; Gonzalez Naranjo LA; Gonzalez Bello YC; Collado MV; Sarano J; Retamozo S; Sattler ME; Gamboa-Cardenas RV; Cairoli E; Conti SM; Amezcua-Guerra LM; Silveira LH; Borba EF; Pera MA; Alba Moreyra PB; Arturi V; Berbotto GA; Gerling C; Gobbi CA; Gervasoni VL; Scherbarth HR; Brenol JCT; Cavalcanti F; Costallat LTL; Da Silva NA; Monticielo

OA; Seguro LPC; Xavier RM; Llanos C; Montufar Guardado RA; Garcia de la Torre I; Pineda C; Portela Hernandez M; Danza A; Guibert-Toledano M; Reyes GL; Acosta Colman MI; Aquino AM; Mora-Trujillo CS; Munoz-Louis R; Garcia Valladares I; Orozco MC; Burgos PI; Betancur GV; Alarcon GS; Grupo Latino Americano de Estudio del Lupus (GLADEL) and Pan-American League of Associations of Rheumatology (PANLAR).

Annals of the Rheumatic Diseases. 77(11):1549-1557, 2018 11.

[Journal Article. Practice Guideline. Research Support, Non-U.S. Gov't]

UI: 30045853

Title Comment
[Comment in: Ann Rheum Dis. 2018 Nov;77(11):1547-1548; PMID: 30309872 [https://www-ncbi-nlm-nih-gov.ezproxy.library.ubc.ca/pub...]]

Authors Full Name
Pons-Estel, Bernardo A; Bonfa, Eloisa; Soriano, Enrique R; Cardiel, Mario H; Izcovich, Ariel; Popoff, Federico; Criniti, Juan M; Vasquez, Gloria; Massardo, Loreto; Duarte, Margarita; Barile-Fabris, Leonor A; Garcia, Mercedes A; Amigo, Mary-Carmen; Espada, Graciela; Catoggio, Luis J; Sato, Emilia Inoue; Levy, Roger A; Acevedo Vasquez, Eduardo M; Chacon-Diaz, Rosa; Galarza-Maldonado, Claudio M; Iglesias Gamarra, Antonio J; Molina, Jose Fernando; Neira, Oscar; Silva, Clovis A; Vargas Pena, Andrea; Gomez-Puerta, Jose A; Scolnik, Marina; Pons-Estel, Guillermo J; Ugolini-Lopes, Michelle R; Savio, Veronica; Drenkard, Cristina; Alvarellos, Alejandro J; Ugarte-Gil, Manuel F; Babini, Alejandra; Cavalcanti, Andre; Cardoso Linhares, Fernanda Athayde; Haye Salinas, Maria Jezabel; Fuentes-Silva, Yurilis J; Montandon de Oliveira E Silva, Ana Carolina; Eraso Garnica, Ruth M; Herrera Uribe, Sebastian; Gomez-Martin, Diana; Robaina Sevrini, Ricardo; Quintana, Rosana M; Gordon, Sergio; Fragoso-Loyo, Hilda; Rosario, Violeta; Saurit, Veronica; Appenzeller, Simone; Dos Reis Neto, Edgard Torres; Cieza, Jorge; Gonzalez Naranjo, Luis A; Gonzalez Bello, Yelitza C; Collado, Maria Victoria; Sarano, Judith; Retamozo, Soledad; Sattler, Maria E; Gamboa-Cardenas, Rocio V; Cairoli, Ernesto; Conti, Silvana M; Amezcua-Guerra, Luis M; Silveira, Luis H; Borba, Eduardo F; Pera, Mariana A; Alba Moreyra, Paula B; Arturi, Valeria; Berbotto, Guillermo A; Gerling, Cristian; Gobbi, Carla A; Gervasoni, Viviana L; Scherbarth, Hugo R; Brenol, Joao C Tavares; Cavalcanti, Fernando; Costallat, Lilian T Lavras; Da Silva, Nilzio A; Monticielo, Odirlei A; Seguro, Luciana Parente Costa; Xavier, Ricardo M; Llanos, Carolina; Montufar Guardado, Ruben A; Garcia de la Torre, Ignacio; Pineda, Carlos; Portela Hernandez, Margarita; Danza, Alvaro; Guibert-Toledano, Marlene; Reyes, Gil Llerena; Acosta Colman, Maria Isabel; Aquino, Alicia M; Mora-Trujillo, Claudia S; Munoz-Louis, Roberto; Garcia Valladares, Ignacio; Orozco, Maria Celeste; Burgos, Paula I; Betancur, Graciela V; Alarcon, Graciela S; Grupo Latino Americano de Estudio del Lupus (GLADEL) and Pan-American League of Associations of Rheumatology (PANLAR).

▶ Abstract + My Projects + Annotate

---

47. **Addiction disorders: a need for change. Proposal for a new management. Position paper of SIA, Italian Society on Alcohol.**

Abstract Reference
Complete Reference

Find Similar
Find Citing Articles

Bibliographic Links
UBC eLink

Testino G; Bottaro LC; Patussi V; Scafato E; Addolorato G; Leone S; Renzetti D; Balbinot P; Greco G; Fanucchi T; Schiappacasse G; Cardinale P; Allosio P; Pellicano R; Caputo F; Study Committee of SIA (Societa Italiana di Alcologia).

Minerva Medica. 109(5):369-385, 2018 Oct.

[Guideline. Journal Article]

UI: 29963833

Authors Full Name
Testino, Gianni; Bottaro, Luigi C; Patussi, Valentino; Scafato, Emanuele; Addolorato, Giovanni; Leone, Silvia; Renzetti, Doda; Balbinot, Patrizia; Greco, Giovanni; Fanucchi, Tiziana; Schiappacasse, Giorgio; Cardinale, Paola; Allosio, Pierluigi; Pellicano, Rinaldo; Caputo, Fabio; Study Committee of SIA (Societa Italiana di Alcologia).

Abstract + My Projects + Annotate

48. **Society of Anesthesia and Sleep Medicine Guideline on Intraoperative Management of Adult Patients With Obstructive Sleep Apnea.**

Memtsoudis SG; Cozowicz C; Nagappa M; Wong J; Joshi GP; Wong DT; Doufas AG; Yilmaz M; Stein MH; Krajewski ML; Singh M; Pichler L; Ramachandran SK; Chung F.

Anesthesia & Analgesia. 127(4):967-987, 2018 10.

[Journal Article. Practice Guideline. Research Support, Non-U.S. Gov't. Systematic Review]

UI: 29944522

Title Comment
[Comment in: Anesth Analg. 2018 Oct;127(4):815-816; PMID: 30216284 [https://www-ncbi-nlm-nih-gov.ezproxy.library.ubc.ca/pub...]]

Authors Full Name
Memtsoudis, Stavros G; Cozowicz, Crispiana; Nagappa, Mahesh; Wong, Jean; Joshi, Girish P; Wong, David T; Doufas, Anthony G; Yilmaz, Meltem; Stein, Mark H; Krajewski, Megan L; Singh, Mandeep; Pichler, Lukas; Ramachandran, Satya Krishna; Chung, Frances.

Abstract Article as PDF (242KB) + My Projects + Annotate

Ovid Full Text
Abstract Reference
Complete Reference

Find Similar
Find Citing Articles

Full Text
Bibliographic Links
UBC eLink

49. **AAPL Practice Resource for Prescribing in Corrections. [Review]**

Anonymous.

Journal of the American Academy of Psychiatry & the Law. 46(2 Supplement):S2-S50, 2018 Jun.

[Journal Article. Practice Guideline. Review]

UI: 29884616

+ My Projects + Annotate

Complete Reference

Find Similar
Find Citing Articles

Full Text
Bibliographic Links
UBC eLink

50. **S3 Guideline for the treatment of psoriasis vulgaris, update - Short version part 2 - Special patient populations and treatment situations.**

Nast A; Amelunxen L; Augustin M; Boehncke WH; Dressler C; Gaskins M; Harle P; Hoffstadt B; Klaus J; Koza J; Mrowietz U; Ockenfels HM; Philipp S; Reich K; Rosenbach T; Rzany B; Schlaeger M; Schmid-Ott G; Sebastian M; von Kiedrowski R; Weberschock T.

Journal der Deutschen Dermatologischen Gesellschaft. 16(6):806-813, 2018 Jun.

[Journal Article. Practice Guideline]

UI: 29873906

Authors Full Name
Nast, Alexander; Amelunxen, Lasse; Augustin, Matthias; Boehncke, Wolf-Henning; Dressler, Corinna; Gaskins, Matthew; Harle, Peter; Hoffstadt, Bernd; Klaus, Joachim; Koza, Joachim; Mrowietz, Ulrich; Ockenfels, Hans-Michael; Philipp, Sandra; Reich, Kristian; Rosenbach, Thomas; Rzany, Berthold; Schlaeger, Martin; Schmid-Ott, Gerhard; Sebastian, Michael; von Kiedrowski, Ralph; Weberschock, Tobias.

Abstract + My Projects + Annotate

Abstract Reference
Complete Reference

Find Similar
Find Citing Articles

Bibliographic Links
UBC eLink

51. **Position Paper for the Treatment of Nightmare Disorder in Adults: An American Academy of Sleep Medicine Position Paper.**

Morgenthaler TI; Auerbach S; Casey KR; Kristo D; Maganti R; Ramar K; Zak R; Kartje R.

Journal of Clinical Sleep Medicine. 14(6):1041-1055, 2018 06 15.

[Journal Article. Practice Guideline. Research Support, Non-U.S. Gov't]

UI: 29852917

Authors Full Name
Morgenthaler, Timothy I; Auerbach, Sanford; Casey, Kenneth R; Kristo, David; Maganti, Rama; Ramar, Kannan; Zak, Rochelle; Kartje, Rebecca.

Abstract + My Projects + Annotate

Abstract Reference
Complete Reference
Find Similar
Find Citing Articles
Full Text
Bibliographic Links
UBC eLink

52. **Practical Assessment and Management of Vulnerabilities in Older Patients Receiving Chemotherapy: ASCO Guideline for Geriatric Oncology.**

Mohile SG; Dale W; Somerfield MR; Schonberg MA; Boyd CM; Burhenn PS; Canin B; Cohen HJ; Holmes HM; Hopkins JO; Janelsins MC; Khorana AA; Klepin HD; Lichtman SM; Mustian KM; Tew WP; Hurria A.

Journal of Clinical Oncology. 36(22):2326-2347, 2018 08 01.

[Journal Article. Practice Guideline. Research Support, N.I.H., Extramural. Research Support, Non-U.S. Gov't]

UI: 29782209

Authors Full Name
Mohile, Supriya G; Dale, William; Somerfield, Mark R; Schonberg, Mara A; Boyd, Cynthia M; Burhenn, Peggy S; Canin, Beverly; Cohen, Harvey Jay; Holmes, Holly M; Hopkins, Judith O; Janelsins, Michelle C; Khorana, Alok A; Klepin, Heidi D; Lichtman, Stuart M; Mustian, Karen M; Tew, William P; Hurria, Arti.

Abstract + My Projects + Annotate

Abstract Reference
Complete Reference
Find Similar
Find Citing Articles
Full Text
Bibliographic Links
UBC eLink

53. **Deprescribing benzodiazepine receptor agonists: Evidence-based clinical practice guideline.**

Pottie K; Thompson W; Davies S; Grenier J; Sadowski CA; Welch V; Holbrook A; Boyd C; Swenson R; Ma A; Farrell B.

Canadian Family Physician. 64(5):339-351, 2018 05.

[Journal Article. Practice Guideline]

UI: 29760253

Authors Full Name
Pottie, Kevin; Thompson, Wade; Davies, Simon; Grenier, Jean; Sadowski, Cheryl A; Welch, Vivian; Holbrook, Anne; Boyd, Cynthia; Swenson, Robert; Ma, Andy; Farrell, Barbara.

Abstract + My Projects + Annotate

Abstract Reference
Complete Reference
Find Similar
Find Citing Articles
Full Text
Bibliographic Links
UBC eLink

54. **Routine Assessment and Promotion of Physical Activity in Healthcare Settings: A Scientific Statement From the American Heart Association. [Review]**

Lobelo F; Rohm Young D; Sallis R; Garber MD; Billinger SA; Duperly J; Hutber

Ovid Full Text
Abstract Reference
Complete Reference

A; Pate RR; Thomas RJ; Widlansky ME; McConnell MV; Joy EA; American Heart Association Physical Activity Committee of the Council on Lifestyle and Cardiometabolic Health; Council on Epidemiology and Prevention; Council on Clinical Cardiology; Council on Genomic and Precision Medicine; Council on Cardiovascular Surgery and Anesthesia; and Stroke Council.

Circulation. 137(18):e495-e522, 2018 05 01.

[Journal Article. Practice Guideline. Review]

UI: 29618598

Authors Full Name
Lobelo, Felipe; Rohm Young, Deborah; Sallis, Robert; Garber, Michael D; Billinger, Sandra A; Duperly, John; Hutber, Adrian; Pate, Russell R; Thomas, Randal J; Widlansky, Michael E; McConnell, Michael V; Joy, Elizabeth A; American Heart Association Physical Activity Committee of the Council on Lifestyle and Cardiometabolic Health; Council on Epidemiology and Prevention; Council on Clinical Cardiology; Council on Genomic and Precision Medicine; Council on Cardiovascular Surgery and Anesthesia; and Stroke Council.

▶ Abstract Article as PDF (837KB) + My Projects + Annotate

Find Similar
Find Citing Articles
Full Text
Bibliographic Links
UBC eLink

---

55. **Canadian Network for Mood and Anxiety Treatments (CANMAT) and International Society for Bipolar Disorders (ISBD) 2018 guidelines for the management of patients with bipolar disorder.**

Yatham LN; Kennedy SH; Parikh SV; Schaffer A; Bond DJ; Frey BN; Sharma V; Goldstein BI; Rej S; Beaulieu S; Alda M; MacQueen G; Milev RV; Ravindran A; O'Donovan C; McIntosh D; Lam RW; Vazquez G; Kapczinski F; McIntyre RS; Kozicky J; Kanba S; Lafer B; Suppes T; Calabrese JR; Vieta E; Malhi G; Post RM; Berk M.

Bipolar Disorders. 20(2):97-170, 2018 03.

[Journal Article. Practice Guideline]

UI: 29536616

Title Comment
[Comment in: Bipolar Disord. 2018 May;20(3):275-276; PMID: 29600547 [https://www-ncbi-nlm-nih-gov.ezproxy.library.ubc.ca/pub...]]
[Comment in: Bipolar Disord. 2018 Jun;20(4):393-394; PMID: 29676513 [https://www-ncbi-nlm-nih-gov.ezproxy.library.ubc.ca/pub...]]

Authors Full Name
Yatham, Lakshmi N; Kennedy, Sidney H; Parikh, Sagar V; Schaffer, Ayal; Bond, David J; Frey, Benicio N; Sharma, Verinder; Goldstein, Benjamin I; Rej, Soham; Beaulieu, Serge; Alda, Martin; MacQueen, Glenda; Milev, Roumen V; Ravindran, Arun; O'Donovan, Claire; McIntosh, Diane; Lam, Raymond W; Vazquez, Gustavo; Kapczinski, Flavio; McIntyre, Roger S; Kozicky, Jan; Kanba, Shigenobu; Lafer, Beny; Suppes, Trisha; Calabrese, Joseph R; Vieta, Eduard; Malhi, Gin; Post, Robert M; Berk, Michael.

▶ Abstract + My Projects + Annotate

Abstract Reference
Complete Reference
Find Similar
Find Citing Articles
Full Text
Bibliographic Links
UBC eLink

---

56. **[New medical treatments for painful endometriosis: CNGOF-HAS Endometriosis Guidelines]. [Review] [French]** Place des nouveaux traitements medicaux dans l'endometriose douloureuse, RPC Endometriose CNGOF-HAS.

Legendre G; Delbos L; Hudon E; Chabbert-Buffet N; Geoffron S; Sauvan M; Fernandez H; Bouet PE; Descamps P.

Gynecologie, Obstetrique, Fertilite & Senologie. 46(3):256-263, 2018 Mar.

[Journal Article. Practice Guideline. Review]

UI: 29530558

Abstract Reference
Complete Reference
Find Similar
Find Citing Articles
Bibliographic Links
UBC eLink

Authors Full Name
Legendre, G; Delbos, L; Hudon, E; Chabbert-Buffet, N; Geoffron, S; Sauvan, M; Fernandez, H; Bouet, P-E; Descamps, P.

▶ Abstract + My Projects + Annotate

---

57. **[Endometriosis medical treatment: Hormonal treatment for the management of pain and endometriotic lesions recurrence. CNGOF-HAS Endometriosis Guidelines]. [Review] [French]** Traitement medical de l'endometriose : prise en charge de la douleur et de l'evolution des lesions par traitement hormonal. RPC Endometriose CNGOF-HAS.

Geoffron S; Cohen J; Sauvan M; Legendre G; Wattier JM; Darai E; Fernandez H; Chabbert-Buffet N.

Gynecologie, Obstetrique, Fertilite & Senologie. 46(3):231-247, 2018 Mar.

[Journal Article. Practice Guideline. Review]

UI: 29530557

Authors Full Name
Geoffron, S; Cohen, J; Sauvan, M; Legendre, G; Wattier, J M; Darai, E; Fernandez, H; Chabbert-Buffet, N.

▶ Abstract + My Projects + Annotate

Abstract Reference
Complete Reference

Find Similar
Find Citing Articles

Bibliographic Links
UBC eLink

---

58. **Guidelines of the International Headache Society for controlled trials of preventive treatment of chronic migraine in adults.**

Tassorelli C; Diener HC; Dodick DW; Silberstein SD; Lipton RB; Ashina M; Becker WJ; Ferrari MD; Goadsby PJ; Pozo-Rosich P; Wang SJ; International Headache Society Clinical Trials Standing Committee.

Cephalalgia. 38(5):815-832, 2018 04.

[Journal Article. Practice Guideline]

UI: 29504482

Authors Full Name
Tassorelli, Cristina; Diener, Hans-Christoph; Dodick, David W; Silberstein, Stephen D; Lipton, Richard B; Ashina, Messoud; Becker, Werner J; Ferrari, Michel D; Goadsby, Peter J; Pozo-Rosich, Patricia; Wang, Shuu-Jiun; International Headache Society Clinical Trials Standing Committee.

▶ Abstract + My Projects + Annotate

Abstract Reference
Complete Reference

Find Similar
Find Citing Articles

Bibliographic Links
UBC eLink

---

59. **Royal Australian and New Zealand College of Psychiatrists clinical practice guidelines for mood disorders: major depression summary.**

Malhi GS; Outhred T; Hamilton A; Boyce PM; Bryant R; Fitzgerald PB; Lyndon B; Mulder R; Murray G; Porter RJ; Singh AB; Fritz K.

Medical Journal of Australia. 208(4):175-180, 2018 03 05.

[Journal Article. Practice Guideline]

UI: 29490210

Authors Full Name
Malhi, Gin S; Outhred, Tim; Hamilton, Amber; Boyce, Philip M; Bryant, Richard; Fitzgerald, Paul B; Lyndon, Bill; Mulder, Roger; Murray, Greg; Porter, Richard J; Singh, Ajeet B; Fritz, Kristina.

▶ Abstract + My Projects + Annotate

Abstract Reference
Complete Reference

Find Similar
Find Citing Articles

Bibliographic Links
UBC eLink

60. **Guidelines for Adolescent Depression in Primary Care (GLAD-PC): Part II. Treatment and Ongoing Management.**

Cheung AH; Zuckerbrot RA; Jensen PS; Laraque D; Stein REK; GLAD-PC STEERING GROUP.

Pediatrics. 141(3), 2018 03.

[Journal Article. Practice Guideline. Research Support, Non-U.S. Gov't]

UI: 29483201

Authors Full Name
Cheung, Amy H; Zuckerbrot, Rachel A; Jensen, Peter S; Laraque, Danielle; Stein, Ruth E K; GLAD-PC STEERING GROUP.

Abstract + My Projects + Annotate

Abstract Reference
Complete Reference
Find Similar
Find Citing Articles
Full Text
Bibliographic Links
UBC eLink

---

61. **Society of Family Planning clinical guidelines pain control in surgical abortion part 1 - local anesthesia and minimal sedation. [Review]**

Allen RH; Singh R.

Contraception. 97(6):471-477, 2018 06.

[Journal Article. Practice Guideline. Review]

UI: 29407363

Authors Full Name
Allen, Rebecca H; Singh, Rameet.

Abstract + My Projects + Annotate

Abstract Reference
Complete Reference
Find Similar
Find Citing Articles
Bibliographic Links
UBC eLink

---

62. **Deprescribing antipsychotics for behavioural and psychological symptoms of dementia and insomnia: Evidence-based clinical practice guideline. [Review]**

Bjerre LM; Farrell B; Hogel M; Graham L; Lemay G; McCarthy L; Raman-Wilms L; Rojas-Fernandez C; Sinha S; Thompson W; Welch V; Wiens A.

Canadian Family Physician. 64(1):17-27, 2018 01.

[Journal Article. Practice Guideline. Review]

UI: 29358245

Authors Full Name
Bjerre, Lise M; Farrell, Barbara; Hogel, Matthew; Graham, Lyla; Lemay, Genevieve; McCarthy, Lisa; Raman-Wilms, Lalitha; Rojas-Fernandez, Carlos; Sinha, Samir; Thompson, Wade; Welch, Vivian; Wiens, Andrew.

Abstract + My Projects + Annotate

Abstract Reference
Complete Reference
Find Similar
Find Citing Articles
Full Text
Bibliographic Links
UBC eLink

---

63. **British Association of Dermatologists' guidelines for the investigation and management of generalized pruritus in adults without an underlying dermatosis, 2018.**

Millington GWM; Collins A; Lovell CR; Leslie TA; Yong ASW; Morgan JD; Ajithkumar T; Andrews MJ; Rushbook SM; Coelho RR; Catten SJ; Lee KYC; Skellett AM; Affleck AG; Exton LS; Mohd Mustapa MF; Levell NJ.

British Journal of Dermatology. 178(1):34-60, 2018 01.

[Journal Article. Practice Guideline]

UI: 29357600

Authors Full Name

Complete Reference
Find Similar
Find Citing Articles
Bibliographic Links
UBC eLink

Millington, G W M; Collins, A; Lovell, C R; Leslie, T A; Yong, A S W; Morgan, J D; Ajithkumar, T; Andrews, M J; Rushbook, S M; Coelho, R R; Catten, S J; Lee, K Y C; Skellett, A M; Affleck, A G; Exton, L S; Mohd Mustapa, M F; Levell, N J.

+ My Projects + Annotate

---

64. **Recommendations of the Spanish Working Group on Crohn's Disease and Ulcerative Colitis (GETECCU) and the Association of Crohn's Disease and Ulcerative Colitis Patients (ACCU) in the management of psychological problems in Inflammatory Bowel Disease patients.** Recomendaciones del Grupo Espanol de Trabajo en Enfermedad de Crohn y Colitis Ulcerosa (GETECCU) y de la Confederacion de Asociaciones de Enfermedad de Crohn y Colitis Ulcerosa (ACCU) para el manejo de los aspectos psicologicos en la enfermedad inflamatoria intestinal.

Abstract Reference
Complete Reference

Find Similar
Find Citing Articles

Bibliographic Links
UBC eLink

Barreiro-de Acosta M; Marin-Jimenez I; Panadero A; Guardiola J; Canas M; Gobbo Montoya M; Modino Y; Alcain G; Bosca-Watts MM; Calvet X; Casellas F; Chaparro M; Fernandez Salazar L; Ferreiro-Iglesias R; Ginard D; Iborra M; Mancenido N; Manosa M; Merino O; Rivero M; Roncero O; Sempere L; Vega P; Zabana Y; Minguez M; Nos P; Gisbert JP.

Gastroenterologia y Hepatologia. 41(2):118-127, 2018 Feb.

[Consensus Development Conference. Journal Article. Practice Guideline. Systematic Review]

UI: 29275001

Authors Full Name
Barreiro-de Acosta, Manuel; Marin-Jimenez, Ignacio; Panadero, Abel; Guardiola, Jordi; Canas, Mercedes; Gobbo Montoya, Milena; Modino, Yolanda; Alcain, Guillermo; Bosca-Watts, Marta Maia; Calvet, Xavier; Casellas, Francesc; Chaparro, Maria; Fernandez Salazar, Luis; Ferreiro-Iglesias, Rocio; Ginard, Daniel; Iborra, Marisa; Mancenido, Noemi; Manosa, Miriam; Merino, Olga; Rivero, Montserrat; Roncero, Oscar; Sempere, Laura; Vega, Pablo; Zabana, Yamile; Minguez, Miguel; Nos, Pilar; Gisbert, Javier P.

▶ Abstract + My Projects + Annotate

---

65. **ENDOCRINE TREATMENT OF GENDER-DYSPHORIC/GENDER-INCONGRUENT PERSONS: AN ENDOCRINE SOCIETY CLINICAL PRACTICE GUIDELINE.**

Complete Reference

Find Similar
Find Citing Articles

Bibliographic Links
UBC eLink

Hembree WC; Cohen-Kettenis PT; Gooren L; Hannema SE; Meyer WJ; Murad MH; Rosenthal SM; Safer JD; Tangpricha V; T'Sjoen GG.

Endocrine Practice. 23(12):1437, 2017 Dec.

[Journal Article. Practice Guideline]

UI: 29320642

Authors Full Name
Hembree, Wylie C; Cohen-Kettenis, Peggy T; Gooren, Louis; Hannema, Sabine E; Meyer, Walter J; Murad, M Hassan; Rosenthal, Stephen M; Safer, Joshua D; Tangpricha, Vin; T'Sjoen, Guy G.

+ My Projects + Annotate

---

66. **Diving and antidepressants. [Review]**

Abstract Reference
Complete Reference

Querido AL.

Diving & Hyperbaric Medicine. 47(4):253-256, 2017 Dec.

[Guideline. Journal Article. Review]

UI: 29241236

Authors Full Name
Querido, Abraham L.

Abstract + My Projects + Annotate

Find Similar
Find Citing Articles

Full Text
Bibliographic Links
UBC eLink

---

67. **2017 HIVMA of IDSA Clinical Practice Guideline for the Management of Chronic Pain in Patients Living With HIV. [Review]**

Bruce RD; Merlin J; Lum PJ; Ahmed E; Alexander C; Corbett AH; Foley K; Leonard K; Treisman GJ; Selwyn P.

Clinical Infectious Diseases. 65(10):e1-e37, 2017 Oct 30.

[Journal Article. Practice Guideline. Review]

UI: 29020263

Title Comment
[Comment in: Clin Infect Dis. 2018 Mar 19;66(7):1152-1153; PMID: 29088381 [https://www-ncbi-nlm-nih-gov.ezproxy.library.ubc.ca/pub...]]

Authors Full Name
Bruce, R Douglas; Merlin, Jessica; Lum, Paula J; Ahmed, Ebtesam; Alexander, Carla; Corbett, Amanda H; Foley, Kathleen; Leonard, Kate; Treisman, Glenn Jordan; Selwyn, Peter.

Abstract + My Projects + Annotate

Abstract Reference
Complete Reference

Find Similar
Find Citing Articles

Full Text
Bibliographic Links
UBC eLink

---

68. **The 2016 Royal Australian and New Zealand College of Psychiatrists guidelines for the management of schizophrenia and related disorders.**

Castle DJ; Galletly CA; Dark F; Humberstone V; Morgan VA; Killackey E; Kulkarni J; McGorry P; Nielssen O; Tran NT; Jablensky A.

Medical Journal of Australia. 206(11):501-505, 2017 Jun 19.

[Guideline. Journal Article]

UI: 28918734

Authors Full Name
Castle, David J; Galletly, Cherrie A; Dark, Frances; Humberstone, Verity; Morgan, Vera A; Killackey, Eoin; Kulkarni, Jayashri; McGorry, Patrick; Nielssen, Olav; Tran, Nga T; Jablensky, Assen.

Abstract + My Projects + Annotate

Abstract Reference
Complete Reference

Find Similar
Find Citing Articles

Bibliographic Links
UBC eLink

---

69. **Endocrine Treatment of Gender-Dysphoric/Gender-Incongruent Persons.**

Radix A; Davis AM.

JAMA. 318(15):1491-1492, 2017 Oct 17.

[Journal Article. Practice Guideline]

UI: 28903155

Authors Full Name
Radix, Asa; Davis, Andrew M.

+ My Projects + Annotate

Complete Reference

Find Similar
Find Citing Articles

Bibliographic Links
UBC eLink

70. **Methodological recommendations for cognition trials in bipolar disorder by the International Society for Bipolar Disorders Targeting Cognition Task Force.**

Abstract Reference
Complete Reference

Find Similar
Find Citing Articles

Full Text
Bibliographic Links
UBC eLink

Miskowiak KW; Burdick KE; Martinez-Aran A; Bonnin CM; Bowie CR; Carvalho AF; Gallagher P; Lafer B; Lopez-Jaramillo C; Sumiyoshi T; McIntyre RS; Schaffer A; Porter RJ; Torres IJ; Yatham LN; Young AH; Kessing LV; Vieta E.

Bipolar Disorders. 19(8):614-626, 2017 12.

[Journal Article. Practice Guideline. Research Support, Non-U.S. Gov't]

UI: 28895274

Title Comment
[Comment in: Bipolar Disord. 2018 Feb;20(1):72-73; PMID: 29214732 [https://www-ncbi-nlm-nih-gov.ezproxy.library.ubc.ca/pub...]]

Authors Full Name
Miskowiak, K W; Burdick, K E; Martinez-Aran, A; Bonnin, C M; Bowie, C R; Carvalho, A F; Gallagher, P; Lafer, B; Lopez-Jaramillo, C; Sumiyoshi, T; McIntyre, R S; Schaffer, A; Porter, R J; Torres, I J; Yatham, L N; Young, A H; Kessing, L V; Vieta, E.

Abstract + My Projects + Annotate

---

71. **European guideline for the diagnosis and treatment of insomnia.**

Abstract Reference
Complete Reference

Find Similar
Find Citing Articles

Bibliographic Links
UBC eLink

Riemann D; Baglioni C; Bassetti C; Bjorvatn B; Dolenc Groselj L; Ellis JG; Espie CA; Garcia-Borreguero D; Gjerstad M; Goncalves M; Hertenstein E; Jansson-Frojmark M; Jennum PJ; Leger D; Nissen C; Parrino L; Paunio T; Pevernagie D; Verbraecken J; Wees HG; Wichniak A; Zavalko I; Arnardottir ES; Deleanu OC; Strazisar B; Zoetmulder M; Spiegelhalder K.

Journal of Sleep Research. 26(6):675-700, 2017 12.

[Journal Article. Practice Guideline]

UI: 28875581

Title Comment
[Comment in: J Sleep Res. 2017 Dec;26(6):701; PMID: 29047179 [https://www-ncbi-nlm-nih-gov.ezproxy.library.ubc.ca/pub...]]

Authors Full Name
Riemann, Dieter; Baglioni, Chiara; Bassetti, Claudio; Bjorvatn, Bjorn; Dolenc Groselj, Leja; Ellis, Jason G; Espie, Colin A; Garcia-Borreguero, Diego; Gjerstad, Michaela; Goncalves, Marta; Hertenstein, Elisabeth; Jansson-Frojmark, Markus; Jennum, Poul J; Leger, Damien; Nissen, Christoph; Parrino, Liborio; Paunio, Tiina; Pevernagie, Dirk; Verbraecken, Johan; Wees, Hans-Gunter; Wichniak, Adam; Zavalko, Irina; Arnardottir, Erna S; Deleanu, Oana-Claudia; Strazisar, Barbara; Zoetmulder, Marielle; Spiegelhalder, Kai.

Abstract + My Projects + Annotate

---

72. **[French Society for Biological Psychiatry and Neuropsychopharmacology and Fondation FondaMental task force: Formal Consensus for the management of treatment-resistant depression]. [French]** Prise en charge des troubles depressifs resistants : recommandations francaises formalisees par des experts de l'AFPBN et de la fondation FondaMental.

Abstract Reference
Complete Reference

Find Similar
Find Citing Articles

Bibliographic Links
UBC eLink

Charpeaud T; Genty JB; Destouches S; Yrondi A; Lancrenon S; Alaili N; Bellivier F; Bennabi D; Bougerol T; Camus V; D'amato T; Doumy O; Haesebaert F; Holtzmann J; Lancon C; Lefebvre M; Moliere F; Nieto I; Richieri R; Schmitt L; Stephan F; Vaiva G; Walter M; Leboyer M; El-Hage W; Haffen E; Llorca PM; Courtet P; Aouizerate B.

Encephale. 43(4S):S1-S24, 2017 Sep.

[Journal Article. Practice Guideline]

UI: 28822460

Authors Full Name
Charpeaud, T; Genty, J-B; Destouches, S; Yrondi, A; Lancrenon, S; Alaili, N; Bellivier, F; Bennabi, D; Bougerol, T; Camus, V; D'amato, T; Doumy, O; Haesebaert, F; Holtzmann, J; Lancon, C; Lefebvre, M; Moliere, F; Nieto, I; Richieri, R; Schmitt, L; Stephan, F; Vaiva, G; Walter, M; Leboyer, M; El-Hage, W; Haffen, E; Llorca, P-M; Courtet, P; Aouizerate, B.

▶ Abstract + My Projects + Annotate

---

73. **[Melatonin - known problems and perspectives of clinical usage]. [Russian]** Melatonin - izvestnye i novye oblasti klinicheskogo primeneniia.

Zakharov AV; Khivintseva EV; Pytin VF; Sergeeva MS; Antipov OI.

Zhurnal Nevrologii i Psikhiatrii Imeni S.S. Korsakova. 117(4. Vyp. 2):74-78, 2017.

[Practice Guideline]

UI: 28777368

Authors Full Name
Zakharov, A V; Khivintseva, E V; Pytin, V F; Sergeeva, M S; Antipov, O I.

▶ Abstract + My Projects + Annotate

Abstract Reference
Complete Reference
Find Similar
Find Citing Articles
Bibliographic Links
UBC eLink

---

74. **Methamphetamine-Related Disorders.**

Gouzoulis-Mayfrank E; Hartel-Petri R; Hamdorf W; Havemann-Reinecke U; Muhlig S; Wodarz N.

Deutsches Arzteblatt International. 114(26):455-461, 2017 Jun 30.

[Journal Article. Practice Guideline]

UI: 28705298

Authors Full Name
Gouzoulis-Mayfrank, Euphrosyne; Hartel-Petri, Roland; Hamdorf, Willem; Havemann-Reinecke, Ursula; Muhlig, Stephan; Wodarz, Norbert.

▶ Abstract + My Projects + Annotate

Abstract Reference
Complete Reference
Find Similar
Find Citing Articles
Full Text
Bibliographic Links
UBC eLink

---

75. **Committee Opinion No. 705: Mental Health Disorders in Adolescents.**

Committee on Adolescent Health Care.

Obstetrics & Gynecology. 130(1):e32-e41, 2017 07.

[Journal Article. Practice Guideline]

UI: 28644337

Authors Full Name
Committee on Adolescent Health Care.

▶ Abstract Article as PDF (360KB) + My Projects
+ Annotate

Ovid Full Text
Abstract Reference
Complete Reference
Find Similar
Find Citing Articles
Bibliographic Links
UBC eLink

---

76. **Committee Opinion No 705 Summary: Mental Health Disorders In Adolescents.**

Anonymous.

Ovid Full Text
Abstract Reference
Complete Reference

Obstetrics & Gynecology. 130(1):247-248, 2017 07.

[Journal Article. Practice Guideline]

UI: 28644330

Abstract Article as PDF (167KB) + My Projects
+ Annotate

Find Similar
Find Citing Articles

Bibliographic Links
UBC eLink

---

77. **European Association for Neuro-Oncology (EANO) guidelines for palliative care in adults with glioma. [Review]**

Pace A; Dirven L; Koekkoek JAF; Golla H; Fleming J; Ruda R; Marosi C; Rhun EL; Grant R; Oliver K; Oberg I; Bulbeck HJ; Rooney AG; Henriksson R; Pasman HRW; Oberndorfer S; Weller M; Taphoorn MJB; European Association of Neuro-Oncology palliative care task force.

Lancet Oncology. 18(6):e330-e340, 2017 06.

[Journal Article. Practice Guideline. Review. Systematic Review]

UI: 28593859

Authors Full Name
Pace, Andrea; Dirven, Linda; Koekkoek, Johan A F; Golla, Heidrun; Fleming, Jane; Ruda, Roberta; Marosi, Christine; Rhun, Emilie Le; Grant, Robin; Oliver, Kathy; Oberg, Ingela; Bulbeck, Helen J; Rooney, Alasdair G; Henriksson, Roger; Pasman, H Roeline W; Oberndorfer, Stefan; Weller, Michael; Taphoorn, Martin J B; European Association of Neuro-Oncology palliative care task force.

Abstract + My Projects + Annotate

Abstract Reference
Complete Reference

Find Similar
Find Citing Articles

Bibliographic Links
UBC eLink

---

78. **World Federation of Societies of Biological Psychiatry (WFSBP) guidelines for biological treatment of schizophrenia - a short version for primary care.**

Hasan A; Falkai P; Wobrock T; Lieberman J; Glenthoj B; Gattaz WF; Thibaut F; Moller HJ; WFSBP Task Force on Treatment Guidelines for Schizophrenia.

International Journal of Psychiatry in Clinical Practice. 21(2):82-90, 2017 Jun.

[Guideline. Journal Article]

UI: 28498090

Authors Full Name
Hasan, Alkomiet; Falkai, Peter; Wobrock, Thomas; Lieberman, Jeffrey; Glenthoj, Birte; Gattaz, Wagner F; Thibaut, Florence; Moller, Hans-Jurgen; WFSBP Task Force on Treatment Guidelines for Schizophrenia.

Abstract + My Projects + Annotate

Abstract Reference
Complete Reference

Find Similar
Find Citing Articles

Bibliographic Links
UBC eLink

---

79. **Interaction of lifestyle, behaviour or systemic diseases with dental caries and periodontal diseases: consensus report of group 2 of the joint EFP/ORCA workshop on the boundaries between caries and periodontal diseases.**

Chapple IL; Bouchard P; Cagetti MG; Campus G; Carra MC; Cocco F; Nibali L; Hujoel P; Laine ML; Lingstrom P; Manton DJ; Montero E; Pitts N; Range H; Schlueter N; Teughels W; Twetman S; Van Loveren C; Van der Weijden F; Vieira AR; Schulte AG.

Journal of Clinical Periodontology. 44 Suppl 18:S39-S51, 2017 Mar.

[Consensus Development Conference. Journal Article. Practice Guideline. Systematic Review]

UI: 28266114

Abstract Reference
Complete Reference

Find Similar
Find Citing Articles

Bibliographic Links
UBC eLink

Authors Full Name
Chapple, Iain L C; Bouchard, Philippe; Cagetti, Maria Grazia; Campus, Guglielmo; Carra, Maria-Clotilde; Cocco, Fabio; Nibali, Luigi; Hujoel, Philippe; Laine, Marja L; Lingstrom, Peter; Manton, David J; Montero, Eduardo; Pitts, Nigel; Range, Helene; Schlueter, Nadine; Teughels, Wim; Twetman, Svante; Van Loveren, Cor; Van der Weijden, Fridus; Vieira, Alexandre R; Schulte, Andreas G.

▶ Abstract + My Projects + Annotate

---

80. **European Association of Urology Position Statement on the Role of the Urologist in the Management of Male Hypogonadism and Testosterone Therapy.**

Mirone V; Debruyne F; Dohle G; Salonia A; Sofikitis N; Verze P; Fode M; Chapple C; URO-TRAM working group.

European Urology. 72(2):164-167, 2017 08.

[Editorial. Practice Guideline]

UI: 28249799

Authors Full Name
Mirone, Vincenzo; Debruyne, Frans; Dohle, Gert; Salonia, Andrea; Sofikitis, Nikolaos; Verze, Paolo; Fode, Mikkel; Chapple, Christopher; URO-TRAM working group.

▶ Abstract + My Projects + Annotate

Abstract Reference
Complete Reference
Find Similar
Find Citing Articles
Bibliographic Links
UBC eLink

---

81. **ACG Clinical Guideline: Preventive Care in Inflammatory Bowel Disease. [Review]**

Farraye FA; Melmed GY; Lichtenstein GR; Kane SV.

American Journal of Gastroenterology. 112(2):241-258, 2017 02.

[Journal Article. Practice Guideline. Review]

UI: 28071656

Title Comment
[Erratum in: Am J Gastroenterol. 2017 Jul;112(7):1208; PMID: 28534523 [https://www-ncbi-nlm-nih-gov.ezproxy.library.ubc.ca/pub...]]
[Comment in: Inflamm Bowel Dis. 2017 Sep;23 (9):E42; PMID: 28816758 [https://www-ncbi-nlm-nih-gov.ezproxy.library.ubc.ca/pub...]]

Authors Full Name
Farraye, Francis A; Melmed, Gil Y; Lichtenstein, Gary R; Kane, Sunanda V.

▶ Abstract Article as PDF (267KB) + My Projects
+ Annotate

Ovid Full Text
Abstract Reference
Complete Reference
Find Similar
Find Citing Articles
Bibliographic Links
UBC eLink

---

82. **Guidelines for the Detection of Babesia and Theileria Parasites.**

Lempereur L; Beck R; Fonseca I; Marques C; Duarte A; Santos M; Zuquete S; Gomes J; Walder G; Domingos A; Antunes S; Baneth G; Silaghi C; Holman P; Zintl A.

Vector Borne & Zoonotic Diseases. 17(1):51-65, 2017 01.

[Journal Article. Practice Guideline]

UI: 28055573

Authors Full Name
Lempereur, Laetitia; Beck, Relja; Fonseca, Isabel; Marques, Catia; Duarte, Ana; Santos, Marcos; Zuquete, Sara; Gomes, Jacinto; Walder, Gernot; Domingos, Ana; Antunes, Sandra; Baneth, Gad; Silaghi, Cornelia; Holman, Patricia; Zintl, Annetta.

Abstract Reference
Complete Reference
Find Similar
Find Citing Articles
Bibliographic Links
UBC eLink

▶ Abstract + My Projects + Annotate

83. **Clinical Pharmacogenetic Testing and Application: Laboratory Medicine Clinical Practice Guidelines.**

Abstract Reference
Complete Reference

Kim S; Yun YM; Chae HJ; Cho HJ; Ji M; Kim IS; Wee KA; Lee W; Song SH; Woo HI; Lee SY; Chun S.

Find Similar
Find Citing Articles

Annals of Laboratory Medicine. 37(2):180-193, 2017 Mar.

[Practice Guideline]

Full Text
Bibliographic Links
UBC eLink

UI: 28029011

Authors Full Name
Kim, Sollip; Yun, Yeo Min; Chae, Hyo Jin; Cho, Hyun Jung; Ji, Misuk; Kim, In Suk; Wee, Kyung A; Lee, Woochang; Song, Sang Hoon; Woo, Hye In; Lee, Soo Youn; Chun, Sail.

▶ Abstract + My Projects + Annotate

84. **Clinical Practice Guideline for the Pharmacologic Treatment of Chronic Insomnia in Adults: An American Academy of Sleep Medicine Clinical Practice Guideline.**

Abstract Reference
Complete Reference

Sateia MJ; Buysse DJ; Krystal AD; Neubauer DN; Heald JL.

Find Similar
Find Citing Articles

Journal of Clinical Sleep Medicine. 13(2):307-349, 2017 Feb 15.

[Journal Article. Practice Guideline]

Full Text
Bibliographic Links
UBC eLink

UI: 27998379

Title Comment
[Comment in: J Clin Sleep Med. 2017 Jun 15;13(6):837; PMID: 28416045 [https://www-ncbi-nlm-nih-gov.ezproxy.library.ubc.ca/pub...]]
[Comment in: J Clin Sleep Med. 2017 Jun 15;13(6):839; PMID: 28454603 [https://www-ncbi-nlm-nih-gov.ezproxy.library.ubc.ca/pub...]]

Authors Full Name
Sateia, Michael J; Buysse, Daniel J; Krystal, Andrew D; Neubauer, David N; Heald, Jonathan L.

▶ Abstract + My Projects + Annotate

85. **AAP Updates Recommendations for Routine Preventive Pediatric Health Care.**

Abstract Reference
Complete Reference

Lambert M.

Find Similar
Find Citing Articles

American Family Physician. 94(4):324, 2016 Aug 15.

[Journal Article. Practice Guideline]

UI: 27548604

Bibliographic Links
UBC eLink

Authors Full Name
Lambert, Mara.

▶ Abstract + My Projects + Annotate

86. **2016 European guideline on Mycoplasma genitalium infections. [Review]**

Abstract Reference
Complete Reference

Jensen JS; Cusini M; Gomberg M; Moi H.

Journal of the European Academy of Dermatology & Venereology. 30(10):1650-1656, 2016 Oct.

[Journal Article. Practice Guideline. Review]

UI: 27505296

Authors Full Name
Jensen, J S; Cusini, M; Gomberg, M; Moi, H.

▶ Abstract + My Projects + Annotate

Find Similar
Find Citing Articles

Bibliographic Links
UBC eLink

---

87. **Management of Depression in Patients With Cancer: A Clinical Practice Guideline.**

Li M; Kennedy EB; Byrne N; Gerin-Lajoie C; Katz MR; Keshavarz H; Sellick S; Green E.

Journal of oncology practice/American Society of Clinical Oncology. 12(8):747-56, 2016 08.

[Journal Article. Practice Guideline. Research Support, Non-U.S. Gov't]

UI: 27382000

Title Comment
[Erratum in: J Oncol Pract. 2017 Feb;13(2):144; PMID: 29429393 [https://www-ncbi-nlm-nih-gov.ezproxy.library.ubc.ca/pub...]]

Authors Full Name
Li, Madeline; Kennedy, Erin B; Byrne, Nelson; Gerin-Lajoie, Caroline; Katz, Mark R; Keshavarz, Homa; Sellick, Scott; Green, Esther.

▶ Abstract + My Projects + Annotate

Abstract Reference
Complete Reference

Find Similar
Find Citing Articles

Full Text
Bibliographic Links
UBC eLink

---

88. **AMERICAN ASSOCIATION OF CLINICAL ENDOCRINOLOGISTS AND AMERICAN COLLEGE OF ENDOCRINOLOGY COMPREHENSIVE CLINICAL PRACTICE GUIDELINES FOR MEDICAL CARE OF PATIENTS WITH OBESITY.**

Garvey WT; Mechanick JI; Brett EM; Garber AJ; Hurley DL; Jastreboff AM; Nadolsky K; Pessah-Pollack R; Plodkowski R; Reviewers of the AACE/ACE Obesity Clinical Practice Guidelines.

Endocrine Practice. 22 Suppl 3:1-203, 2016 Jul.

[Journal Article. Practice Guideline]

UI: 27219496

Authors Full Name
Garvey, W Timothy; Mechanick, Jeffrey I; Brett, Elise M; Garber, Alan J; Hurley, Daniel L; Jastreboff, Ania M; Nadolsky, Karl; Pessah-Pollack, Rachel; Plodkowski, Raymond; Reviewers of the AACE/ACE Obesity Clinical Practice Guidelines.

▶ Abstract + My Projects + Annotate

Abstract Reference
Complete Reference

Find Similar
Find Citing Articles

Bibliographic Links
UBC eLink

---

89. **BAP guidelines on the management of weight gain, metabolic disturbances and cardiovascular risk associated with psychosis and antipsychotic drug treatment.**

Cooper SJ; Reynolds GP; With expert co-authors (in alphabetical order):; Barnes T; England E; Haddad PM; Heald A; Holt R; Lingford-Hughes A; Osborn D; McGowan O; Patel MX; Paton C; Reid P; Shiers D; Smith J.

Journal of Psychopharmacology. 30(8):717-48, 2016 08.

[Journal Article. Practice Guideline. Research Support, Non-U.S. Gov't]

Abstract Reference
Complete Reference

Find Similar
Find Citing Articles

Bibliographic Links

UI: 27147592

Authors Full Name

Cooper, Stephen J; Reynolds, Gavin P; With expert co-authors (in alphabetical order):; Barnes, Tre; England, E; Haddad, P M; Heald, A; Holt, Rig; Lingford-Hughes, A; Osborn, D; McGowan, O; Patel, M X; Paton, C; Reid, P; Shiers, D; Smith, J.

▶ Abstract + My Projects + Annotate

UBC eLink

---

90. **Management of Chronic Insomnia Disorder in Adults: A Clinical Practice Guideline From the American College of Physicians.**

Qaseem A; Kansagara D; Forciea MA; Cooke M; Denberg TD; Clinical Guidelines Committee of the American College of Physicians.

Annals of Internal Medicine. 165(2):125-33, 2016 Jul 19.

[Journal Article. Practice Guideline]

UI: 27136449

Title Comment

[Summary for Patients in: Ann Intern Med. 2016 Jul 19;165(2):; PMID: 27135191 [https://www-ncbi-nlm-nih-gov.ezproxy.library.ubc.ca/pub...]]
[Comment in: Ann Intern Med. 2016 Dec 20;165(12):892; PMID: 27992908 [https://www-ncbi-nlm-nih-gov.ezproxy.library.ubc.ca/pub...]]
[Comment in: Ann Intern Med. 2016 Dec 20;165(12):891-892; PMID: 27992909 [https://www-ncbi-nlm-nih-gov.ezproxy.library.ubc.ca/pub...]]

Authors Full Name

Qaseem, Amir; Kansagara, Devan; Forciea, Mary Ann; Cooke, Molly; Denberg, Thomas D; Clinical Guidelines Committee of the American College of Physicians.

▶ Abstract + My Projects + Annotate

Abstract Reference
Complete Reference

Find Similar
Find Citing Articles

Bibliographic Links
UBC eLink

---

91. **The American Psychiatric Association Practice Guideline on the Use of Antipsychotics to Treat Agitation or Psychosis in Patients With Dementia.**

Reus VI; Fochtmann LJ; Eyler AE; Hilty DM; Horvitz-Lennon M; Jibson MD; Lopez OL; Mahoney J; Pasic J; Tan ZS; Wills CD; Rhoads R; Yager J.

American Journal of Psychiatry. 173(5):543-6, 2016 05 01.

[Journal Article. Practice Guideline]

UI: 27133416

Authors Full Name

Reus, Victor I; Fochtmann, Laura J; Eyler, A Evan; Hilty, Donald M; Horvitz-Lennon, Marcela; Jibson, Michael D; Lopez, Oscar L; Mahoney, Jane; Pasic, Jagoda; Tan, Zaldy S; Wills, Cheryl D; Rhoads, Richard; Yager, Joel.

+ My Projects + Annotate

Complete Reference

Find Similar
Find Citing Articles

Bibliographic Links
UBC eLink

---

92. **Royal Australian and New Zealand College of Psychiatrists clinical practice guidelines for the management of schizophrenia and related disorders.**

Galletly C; Castle D; Dark F; Humberstone V; Jablensky A; Killackey E; Kulkarni J; McGorry P; Nielssen O; Tran N.

Australian & New Zealand Journal of Psychiatry. 50(5):410-72, 2016 May.

[Journal Article. Practice Guideline. Research Support, Non-U.S. Gov't]

Abstract Reference
Complete Reference

Find Similar
Find Citing Articles

Bibliographic Links

UI: 27106681

Title Comment
[Comment in: Aust N Z J Psychiatry. 2017 Mar;51(3):287-288; PMID: 27687777 [https://www-ncbi-nlm-nih-gov.ezproxy.library.ubc.ca/pub...]]
[Comment in: Aust N Z J Psychiatry. 2017 Jul;51(7):736-737; PMID: 27687779 [https://www-ncbi-nlm-nih-gov.ezproxy.library.ubc.ca/pub...]]
[Comment in: Aust N Z J Psychiatry. 2017 Mar;51(3):288-289; PMID: 28218054 [https://www-ncbi-nlm-nih-gov.ezproxy.library.ubc.ca/pub...]]
[Comment in: Aust N Z J Psychiatry. 2017 Mar;51(3):289-290; PMID: 28218055 [https://www-ncbi-nlm-nih-gov.ezproxy.library.ubc.ca/pub...]]
[Comment in: Australas Psychiatry. 2019 Jun;27(3):314; PMID: 31189366 [https://www-ncbi-nlm-nih-gov.ezproxy.library.ubc.ca/pub...]]

Authors Full Name
Galletly, Cherrie; Castle, David; Dark, Frances; Humberstone, Verity; Jablensky, Assen; Killackey, Eoin; Kulkarni, Jayashri; McGorry, Patrick; Nielssen, Olav; Tran, Nga.

▶ Abstract + My Projects + Annotate

UBC eLink

---

93. **Nonpharmacologic Versus Pharmacologic Treatment of Adult Patients With Major Depressive Disorder: A Clinical Practice Guideline From the American College of Physicians.**

Qaseem A; Barry MJ; Kansagara D; Clinical Guidelines Committee of the American College of Physicians.

Annals of Internal Medicine. 164(5):350-9, 2016 Mar 01.

[Journal Article. Practice Guideline]

UI: 26857948

Title Comment
[Summary for Patients in: Ann Intern Med. 2016 Mar 1;164(5). doi: 10.7326/P16-9010; PMID: 26856417 [https://www-ncbi-nlm-nih-gov.ezproxy.library.ubc.ca/pub...]]

Authors Full Name
Qaseem, Amir; Barry, Michael J; Kansagara, Devan; Clinical Guidelines Committee of the American College of Physicians.

▶ Abstract + My Projects + Annotate

Abstract Reference
Complete Reference

Find Similar
Find Citing Articles

Bibliographic Links
UBC eLink

---

94. **Management of resistant hypertension: expert consensus statement from the French Society of Hypertension, an affiliate of the French Society of Cardiology. [Review]**

Denolle T; Chamontin B; Doll G; Fauvel JP; Girerd X; Herpin D; Vaisse B; Villeneuve F; Halimi JM.

Journal of Human Hypertension. 30(11):657-663, 2016 11.

[Journal Article. Practice Guideline. Review]

UI: 26818804

Authors Full Name
Denolle, T; Chamontin, B; Doll, G; Fauvel, J-P; Girerd, X; Herpin, D; Vaisse, B; Villeneuve, F; Halimi, J M.

▶ Abstract + My Projects + Annotate

Abstract Reference
Complete Reference

Find Similar
Find Citing Articles

Bibliographic Links
UBC eLink

---

95. **Drugs for behavior disorders after traumatic brain injury: Systematic review and expert consensus leading to French recommendations**

Abstract Reference
Complete Reference

for good practice. [Review]

Plantier D; Luaute J; SOFMER group.

Annals of Physical & Rehabilitation Medicine. 59(1):42-57, 2016 Feb.

[Journal Article. Practice Guideline. Research Support, Non-U.S. Gov't. Review. Systematic Review]

UI: 26797170

Authors Full Name
Plantier, D; Luaute, J; SOFMER group.

▶ Abstract + My Projects + Annotate

Find Similar
Find Citing Articles

Bibliographic Links
UBC eLink

---

96. **Care management of the agitation or aggressiveness crisis in patients with TBI. Systematic review of the literature and practice recommendations. [Review]**

Luaute J; Plantier D; Wiart L; Tell L; SOFMER group.

Annals of Physical & Rehabilitation Medicine. 59(1):58-67, 2016 Feb.

[Journal Article. Practice Guideline. Research Support, Non-U.S. Gov't. Review. Systematic Review]

UI: 26700025

Authors Full Name
Luaute, Jacques; Plantier, David; Wiart, Laurent; Tell, Laurence; SOFMER group.

▶ Abstract + My Projects + Annotate

Abstract Reference
Complete Reference

Find Similar
Find Citing Articles

Bibliographic Links
UBC eLink

---

97. **American Cancer Society/American Society of Clinical Oncology Breast Cancer Survivorship Care Guideline. [Review]**

Runowicz CD; Leach CR; Henry NL; Henry KS; Mackey HT; Cowens-Alvarado RL; Cannady RS; Pratt-Chapman ML; Edge SB; Jacobs LA; Hurria A; Marks LB; LaMonte SJ; Warner E; Lyman GH; Ganz PA.

Journal of Clinical Oncology. 34(6):611-35, 2016 Feb 20.

[Journal Article. Practice Guideline. Research Support, U.S. Gov't, P.H.S.. Review. Systematic Review]

UI: 26644543

Authors Full Name
Runowicz, Carolyn D; Leach, Corinne R; Henry, N Lynn; Henry, Karen S; Mackey, Heather T; Cowens-Alvarado, Rebecca L; Cannady, Rachel S; Pratt-Chapman, Mandi L; Edge, Stephen B; Jacobs, Linda A; Hurria, Arti; Marks, Lawrence B; LaMonte, Samuel J; Warner, Ellen; Lyman, Gary H; Ganz, Patricia A.

▶ Abstract + My Projects + Annotate

Abstract Reference
Complete Reference

Find Similar
Find Citing Articles

Bibliographic Links
UBC eLink

---

98. **Florida Best Practice Psychotherapeutic Medication Guidelines for Adults With Bipolar Disorder: A Novel, Practical, Patient-Centered Guide for Clinicians.**

Ostacher MJ; Tandon R; Suppes T.

Journal of Clinical Psychiatry. 77(7):920-6, 2016 07.

[Guideline. Journal Article. Research Support, Non-U.S. Gov't]

UI: 26580001

Authors Full Name
Ostacher, Michael J; Tandon, Rajiv; Suppes, Trisha.

Abstract Reference
Complete Reference

Find Similar
Find Citing Articles

Bibliographic Links
UBC eLink

▶ Abstract + My Projects + Annotate

99. **Treatment of adult patients with schizophrenia and complex mental health needs - A national clinical guideline.**

Baandrup L; Ostrup Rasmussen J; Klokker L; Austin S; Bjornshave T; Fuglsang Bliksted V; Fink-Jensen A; Hedegaard Fohlmann A; Peter Hansen J; Kristine Nielsen M; Sandsten KE; Schultz V; Voss-Knude S; Nordentoft M.

Nordic Journal of Psychiatry. 70(3):231-40, 2016.

[Journal Article. Practice Guideline. Research Support, Non-U.S. Gov't]

UI: 26328910

Authors Full Name
Baandrup, Lone; Ostrup Rasmussen, Jesper; Klokker, Louise; Austin, Stephen; Bjornshave, Thomas; Fuglsang Bliksted, Vibeke; Fink-Jensen, Anders; Hedegaard Fohlmann, Allan; Peter Hansen, Jens; Kristine Nielsen, Malene; Sandsten, Karl Erik; Schultz, Vilhelm; Voss-Knude, Susanne; Nordentoft, Merete.

▶ Abstract + My Projects + Annotate

Abstract Reference
Complete Reference
Find Similar
Find Citing Articles
Bibliographic Links
UBC eLink

100. **Pharmacotherapy Treatment Options for Insomnia: A Primer for Clinicians. [Review]**

Asnis GM; Thomas M; Henderson MA.

International Journal of Molecular Sciences. 17(1), 2015 Dec 30.

[Journal Article. Practice Guideline. Review]

UI: 26729104

Authors Full Name
Asnis, Gregory M; Thomas, Manju; Henderson, Margaret A.

▶ Abstract + My Projects + Annotate

Abstract Reference
Complete Reference
Find Similar
Find Citing Articles
Full Text
Bibliographic Links
UBC eLink

All Range Clear 100 Per Page 1 Go Next ›

Print Email Export + My Projects Keep Selected

© 2020 Ovid Technologies, Inc. All rights reserved. OvidUI_04.04.00.037, SourceID 7eebd4b

Search History (16)

# ▲	Searches	Results	Type	Actions	Annotations
1	mental disease/ or adjustment disorder/ or alexithymia/ or exp anxiety disorder/ or exp autism/ or exp behavior disorder/ or complicated grief/ or exp confusion/ or exp delirium/ or exp dissociative disorder/ or emotional disorder/ or exp mental deficiency/ or mental instability/ or exp mood disorder/ or exp neurosis/ or exp personality disorder/ or exp psychosexual disorder/ or exp psychosis/ or exp psychosomatic disorder/ or psychotrauma/ or exp schizophrenia spectrum disorder/ or stupor/ or exp thought disorder/	1885967	Advanced	Display Results | More	
2	((psychiatric or mental or behavio?r or anxiety or bipolar or dissociative or mood) adj3 (disorder* or diagnos#s or illness* or disease*)).mp. [mp=title, abstract, heading word, drug trade name, original title, device manufacturer, drug manufacturer, device trade name, keyword, floating subheading word, candidate term word]	543288	Advanced	Display Results | More	
3	(insomnia or depression or mood or bipolar or mania or anxiety or post-traumatic stress disorder* or psychos#s or schizo*).mp. [mp=title, abstract, heading word, drug trade name, original title, device manufacturer, drug manufacturer, device trade name, keyword, floating subheading word, candidate term word]	1205254	Advanced	Display Results | More	
4	1 or 2 or 3	2321508	Advanced	Display Results | More	
5	drug dependence/ or exp alcoholism/ or amphetamine dependence/ or benzodiazepine dependence/ or cannabis addiction/ or cocaine dependence/ or congenital drug dependence/ or drug abuse pattern/ or drug craving/ or exp drug misuse/ or drug seeking behavior/ or glue sniffing/ or methamphetamine dependence/ or multiple drug abuse/ or exp narcotic dependence/ or phencyclidine dependence/ or tobacco dependence/	228994	Advanced	Display Results | More	
6	((drug or substance or alcohol or tobacco or nicotine or marijuana or cannabis) adj3 (depend* or abuse or misuse)).mp. [mp=title, abstract, heading word, drug trade name, original title, device manufacturer, drug manufacturer, device trade name, keyword, floating subheading word, candidate term word]	272350	Advanced	Display Results | More	
7	(heroin or stimulant or crystal meth or methamphetamine or MDMA or GHP or club drugs or LSD or PCP or depressant* or stimulant* or hallucinogen* or prescription narcotic*).mp. [mp=title, abstract, heading word, drug trade name, original title, device manufacturer, drug manufacturer, device trade name, keyword, floating subheading word, candidate term word]	122350	Advanced	Display Results | More	
8	5 or 6 or 7	461432	Advanced	Display Results | More	
9	(concurrent or dual diagnosis or co-occuring or co-morbidity or co-existing or coinciding).mp. [mp=title, abstract, heading word, drug trade name, original title, device manufacturer, drug manufacturer, device trade	201855	Advanced	Display Results | More	

name, keyword, floating subheading word, candidate term word]

10	4 and 9	29949	Advanced	Display Results | More	
11	8 and 9	8996	Advanced	Display Results | More	
12	10 or 11	33706	Advanced	Display Results | More	
13	exp practice guideline/	537827	Advanced	Display Results | More	
14	(guide* or guideline* or best practice or recommendation*).mp. [mp=title, abstract, heading word, drug trade name, original title, device manufacturer, drug manufacturer, device trade name, keyword, floating subheading word, candidate term word]	1429770	Advanced	Display Results | More	
15	13 or 14	1536359	Advanced	Display Results | More	
16	12 and 15	2283	Advanced	Display Results | More	

Save Remove Combine with: AND OR

Save All Edit Create RSS View Saved

Basic Search | Find Citation | Search Tools | Search Fields | **Advanced Search** | Multi-Field Search

1 Resource selected | Hide | Change

Embase 1974 to 2020 March 23

Enter keyword or phrase (* or $ for truncation)

Keyword Author Title Journal

Search

Limits *(expand)* Include Multimedia Map Term to Subject Heading

Options

To search Open Access content on Ovid, go to Basic Search.

Print Email Export + My Projects Keep Selected

All Range Clear 100 Per Page 1 Go Next ›

Search Information

You searched:

12 and 15

Search terms used:

abuse
adjustment
disorder
alcohol
alcoholism
alexithymia
amphetamine
dependence
anxiety
autism
behavio?r
behavior
benzodiazepine
best
practice
bipolar
cannabis
addiction
club
drugs
co-existing
co-morbidity

1. **Physician decision-making and recommendations for stroke and myocardial infarction treatments in older adults with mild cognitive impairment.**

Levine D.A., Langa K.M., Fagerlin A., Morgenstern L.B., Nallamothu B.K., Forman J., Galecki A., Kabeto M.U., Kollman C.D., Olorode T., Giordani B., Lisabeth L.D., Zahuranec D.B.

PLoS ONE. 15 (3) (no pagination), 2020. Article Number: e0230446. Date of Publication: 2020.

[Article]

Publisher
Public Library of Science (E-mail: plos@plos.org)

Abstract + My Projects + Annotate

Abstract Reference
Complete Reference

Find Similar
Find Citing Articles

Full Text
Bibliographic Links
UBC eLink

2. **Best Practices in the Management of Nonmedical Opioid Use in Patients with Cancer-Related Pain.**

Ulker E., Del Fabbro E.

Oncologist. 25 (3) (pp 189-196), 2020. Date of Publication: 01 Mar 2020.

Abstract Reference
Complete Reference

Find Similar

co-occuring
cocaine
coinciding
complicated
grief
concurrent
confusion
congenital
drug
crystal
meth
delirium
depend*
depressant*
depression
diagnos#s
disease*
disorder*
dissociative
pattern
craving
misuse
seeking
dual
diagnosis
emotional
ghp
glue
sniffing
guide*
guideline*
hallucinogen*
heroin
illness*
insomnia
lsd
mania
marijuana
mdma
mental
deficiency
disease
instability
methamphetamine
mood
multiple
narcotic
neurosis
nicotine
pcp
personality
phencyclidine
post-traumatic
stress
guideline
prescription
narcotic*
psychiatric
psychos#s
psychosexual
psychosis
psychosomatic
psychotrauma
recommendation*
schizo*
schizophrenia

[Review]

Publisher
Wiley-Blackwell (E-mail: info@wiley.com)

▶ Abstract + My Projects + Annotate

Find Citing Articles
Full Text
Bibliographic Links
UBC eLink

---

3. **A case of oculopharyngeal muscular dystrophy presenting as dysphagia.**

Varma A.V., England J.

Journal of Investigative Medicine. Conference: 2020 Southern Regional Meeting. United States. 68 (2) (pp 655), 2020. Date of Publication: February 2020.

[Conference Abstract]

Publisher
BMJ Publishing Group

▶ Abstract + My Projects + Annotate

Abstract Reference
Complete Reference
Find Similar
Find Citing Articles
Bibliographic Links
UBC eLink

---

4. **Infected femoral pseudoaneurysms in intravenous drug abusers: a decade of experience from a Singapore tertiary centre.**

Samarakoon L.B., Ho D.C.Y., Tan Y.K., Kum S.W.C., Lim D.M.

Singapore medical journal. (no pagination), 2020. Date of Publication: 09 Mar 2020.

[Article]

Publisher
NLM (Medline)

▶ Abstract + My Projects + Annotate

Abstract Reference
Complete Reference
Find Similar
Find Citing Articles
UBC eLink

---

5. **A randomised controlled trial of hearing and vision support in dementia: Protocol for a process evaluation in the SENSE-Cog trial.**

Armitage C.J., Abrams H., Chaghil-Boissiere N., Charalambous A.P., Collin F., Constantinidou F., Crosby L., Dawes P., Frison E., Gilbert C., Hann M., Hooper E., Himmelsbach I., Kontogianni E., Lawlor B., Leroi I., Marie S., Montecelo S., Politis A., Postea O., Reeves D., Renaud D., Simkin Z., Termote M., Thodi C., Wolski L.

Trials. 21 (1) (no pagination), 2020. Article Number: 223. Date of Publication: 24 Feb 2020.

[Article]

Publisher
BioMed Central Ltd. (E-mail: info@biomedcentral.com)

▶ Abstract + My Projects + Annotate

Abstract Reference
Complete Reference
Find Similar
Find Citing Articles
Full Text
Bibliographic Links
UBC eLink

---

6. **Switch to dolutegravir is well tolerated in Thais with HIV infection.**

Goh O.Q., Colby D.J., Pinyakorn S., Sacdalan C., Kroon E., Chan P., Chomchey N., Kanaprach R., Prueksakaew P., Suttichom D., Trichavaroj R., Spudich S., Robb M.L., Phanuphak P., Phanuphak N., Ananworanich J.

Journal of the International AIDS Society. 22 (7) (no pagination), 2019. Article Number: e25324. Date of Publication: 01 Jul 2019.

Abstract Reference
Complete Reference
Find Similar
Find Citing Articles

spectrum
stimulant
stimulant*
stupor
substance
thought
tobacco

Search Returned:
2283 text results

Sort By:
-

Customize Display

Filter By

Add to Search History

Selected Only (0)

Years
All Years
Current year
Past 3 years
Past 5 years
Specific Year Range

Subject
Author
Journal
Publication Type

My Projects

+ New Project

No projects available.

JBI EBP Tools

SUMARI

[Article]

Publisher
John Wiley and Sons Inc. (E-mail: cs-journals@wiley.com)

Abstract + My Projects + Annotate

Full Text
Bibliographic Links
UBC eLink

---

7. **ADDRESSING DEPRESSION WITH AND WITHOUT COEXISTING ANXIETY IN COMMUNITY-DWELLING PERSONS WITH DEMENTIA.**

Sibley A., Shrestha S., Lipovac-Dew M., Kunik M.

American Journal of Geriatric Psychiatry. Conference: 2020 AAGP Annual Meeting. Grand Hyatt San Antonio, United States. 28 (4 Supplement) (pp S84-S85), 2020. Date of Publication: April 2020.

[Conference Abstract]

Publisher
Elsevier B.V.

Abstract + My Projects + Annotate

Abstract Reference
Complete Reference
Find Similar
Find Citing Articles
Bibliographic Links
UBC eLink

---

8. **Emergency Department Visits Attributed to Adverse Events Involving Benzodiazepines, 2016-2017.**

Moro R.N., Geller A.I., Weidle N.J., Lind J.N., Lovegrove M.C., Rose K.O., Goring S.K., McAninch J.K., Dowell D., Budnitz D.S.

American Journal of Preventive Medicine. 58 (4) (pp 526-535), 2020. Date of Publication: April 2020.

[Article]

Publisher
Elsevier Inc. (E-mail: usjcs@elsevier.com)

Abstract + My Projects + Annotate

Abstract Reference
Complete Reference
Find Similar
Find Citing Articles
Bibliographic Links
UBC eLink

---

9. **Evening chronotype is associated with poor cardiovascular health and adverse health behaviors in a diverse population of women.**

Makarem N., Paul J., Giardina E.-G.V., Liao M., Aggarwal B.

Chronobiology international. (pp 1-13), 2020. Date of Publication: 04 Mar 2020.

[Article]

Publisher
NLM (Medline)

Abstract + My Projects + Annotate

Abstract Reference
Complete Reference
Find Similar
Find Citing Articles
UBC eLink

---

10. **Treatment for substance use disorder with co-occurring mental illness.**

Iqbal M.N., Levin C.J., Levin F.R.

Focus (United States). 17 (2) (pp 88-97), 2019. Date of Publication: 2019.

[Review]

Publisher
American Psychiatric Association

Abstract + My Projects + Annotate

Abstract Reference
Complete Reference
Find Similar
Find Citing Articles
Bibliographic Links
UBC eLink

11. **The Impact of Concurrent Antiretroviral Therapy and MDR-TB Treatment on Adverse Events.**

Smith J.P., Gandhi N.R., Shah N.S., Mlisana K., Moodley P., Johnson B.A., Allana S., Campbell A., Nelson K.N., Master I., Brust J.C.M.

Journal of Acquired Immune Deficiency Syndromes. 83 (1) (pp 47-55), 2020. Date of Publication: 01 Jan 2020.

[Article]

Publisher
Lippincott Williams and Wilkins (E-mail: kathiest.clai@apta.org)

Abstract Article as PDF (382KB) + My Projects + Annotate

Ovid Full Text
Abstract Reference
Complete Reference

Find Similar
Find Citing Articles

Bibliographic Links
UBC eLink

12. **Improving Care Provision to Older Adults with Dual Diagnosis: Recommendations from a Mixed-Methods Study.**

Searby A., Maude P., McGrath I.

Issues in mental health nursing. 41 (3) (pp 229-234), 2020. Date of Publication: 01 Mar 2020.

[Article]

Publisher
NLM (Medline)

Abstract + My Projects + Annotate

Abstract Reference
Complete Reference

Find Similar
Find Citing Articles

Bibliographic Links
UBC eLink

13. **Treatment of substance use disorders with co-occurring severe mental health disorders.**

Murthy P., Mahadevan J., Chand P.K.

Current Opinion in Psychiatry. 32 (4) (pp 293-299), 2019. Date of Publication: 01 Jul 2019.

[Review]

Publisher
Lippincott Williams and Wilkins (E-mail: agents@lww.com)

Abstract Article as PDF (268KB) + My Projects + Annotate

Ovid Full Text
Abstract Reference
Complete Reference

Find Similar
Find Citing Articles

Bibliographic Links
UBC eLink

14. **Risk factors and outcomes of opioid users with and without concurrent benzodiazepine use in the North Carolina medicaid population.**

Hung A., Bush C., Greiner M., Campbell H., Hammill B., MacIejewski M.L., McKethan A.

Journal of Managed Care and Specialty Pharmacy. 26 (2) (pp 169-175), 2020. Date of Publication: 01 Feb 2020.

[Article]

Publisher
Academy of Managed Care Pharmacy (AMCP)

Abstract + My Projects + Annotate

Abstract Reference
Complete Reference

Find Similar
Find Citing Articles

Bibliographic Links
UBC eLink

15. **Pregabalin Abuse in Combination With Other Drugs: Monitoring Among Methadone Patients.**

Lancia M., Gambelunghe A., Gili A., Bacci M., Aroni K., Gambelunghe C.

Frontiers in Psychiatry. 10 (no pagination), 2020. Article Number: 1022. Date of Publication: 11 Feb 2020.

[Article]

Publisher
Frontiers Media S.A. (E-mail: info@frontiersin.org)

▶ Abstract + My Projects + Annotate

Abstract Reference
Complete Reference
Find Similar
Find Citing Articles
Bibliographic Links
UBC eLink

---

16. **Resurgence of Syphilis in the United States: An Assessment of Contributing Factors.**

Schmidt R., Carson P.J., Jansen R.J.

Infectious Diseases: Research and Treatment. 12 (no pagination), 2019. Date of Publication: 2019.

[Review]

Publisher
SAGE Publications Inc. (E-mail: claims@sagepub.com)

▶ Abstract + My Projects + Annotate

Abstract Reference
Complete Reference
Find Similar
Find Citing Articles
UBC eLink

---

17. **The Ohio substance abuse and mental illness coordinating center of excellence.**

Ronis R.J.

Journal of Dual Diagnosis. 1 (1) (pp 107-113), 2005. Date of Publication: 2005.

[Review]

Publisher
Haworth Press Inc.

▶ Abstract + My Projects + Annotate

Abstract Reference
Complete Reference
Find Similar
Find Citing Articles
Bibliographic Links
UBC eLink

---

18. **Developing welcoming systems for individuals with co-occurring disorders: The role of the comprehensive continuous integrated system of care model.**

Minkoff K., Cline C.A.

Journal of Dual Diagnosis. 1 (1) (pp 65-89), 2005. Date of Publication: 2005.

[Review]

Publisher
Haworth Press Inc.

▶ Abstract + My Projects + Annotate

Abstract Reference
Complete Reference
Find Similar
Find Citing Articles
Bibliographic Links
UBC eLink

---

19. **How Do Common Comorbidities Modify the Association of Frailty With Survival After Elective Noncardiac Surgery? A Population-Based Cohort Study.**

Hui Y., Van Walraven C., Mcisaac D.I.

Anesthesia and Analgesia. 129 (6) (pp 1699-1706), 2019. Date of Publication: 01

Ovid Full Text
Abstract Reference
Complete Reference
Find Similar

Dec 2019.

[Article]

Publisher
Lippincott Williams and Wilkins (E-mail: kathiest.clai@apta.org)

▶ Abstract Article as PDF (293KB) + My Projects
+ Annotate

Find Citing Articles

Bibliographic Links
UBC eLink

---

20. **Obesity in children and young people: A crisis in public health.**

Lobstein T., Baur L., Uauy R.

Obesity Reviews, Supplement. 5 (1) (pp 4-85), 2004. Date of Publication: May 2004.

[Review]

Publisher
Blackwell Publishing Ltd

▶ Abstract + My Projects + Annotate

Abstract Reference
Complete Reference

Find Similar
Find Citing Articles

Bibliographic Links
UBC eLink

---

21. **Neuropsychiatric complications of liver and other solid organ transplantation.**

Beresford T.P.

Liver Transplantation. 7 (11 SUPPL. 1) (pp s36-s45), 2001. Date of Publication: 2001.

[Article]

Publisher
W.B. Saunders

▶ Abstract + My Projects + Annotate

Abstract Reference
Complete Reference

Find Similar
Find Citing Articles

Bibliographic Links
UBC eLink

---

22. **Emotion-Focused Therapy: A Transdiagnostic Formulation.**

Timulak L., Keogh D.

Journal of Contemporary Psychotherapy. 50 (1) (no pagination), 2020. Date of Publication: 01 Mar 2020.

[Article]

Publisher
Springer

▶ Abstract + My Projects + Annotate

Abstract Reference
Complete Reference

Find Similar
Find Citing Articles

Bibliographic Links
UBC eLink

---

23. **A conceptual model for co-occurring mental and substance-related disorders.**

Singer M.I., Kennedy M.J., Kola L.A.

Alcoholism Treatment Quarterly. 16 (4) (pp 75-89), 1999. Date of Publication: 1999.

[Article]

Publisher
Haworth Press Inc.

▶ Abstract + My Projects + Annotate

Abstract Reference
Complete Reference

Find Similar
Find Citing Articles

Bibliographic Links
UBC eLink

24. **Gambling and Cannabis Use: Clinical and Policy Implications.**

Winters K.C., Whelan J.P.

Journal of gambling studies. 36 (1) (pp 223-241), 2020. Date of Publication: 01 Mar 2020.

[Review]

Publisher
NLM (Medline)

Abstract + My Projects + Annotate

Abstract Reference
Complete Reference

Find Similar
Find Citing Articles

Bibliographic Links
UBC eLink

25. **A novel and practical method of performing atrioventricular nodal ablation via a superior approach in patients with refractory atrial fibrillation undergoing cardiac resynchronization device implantation.**

Acosta H., Viafara L.M., Hanif N., Acosta S., Pagadala M., Acosta B., Pothula S., Peckosh C., Bear J., Alzate S., Ballesteros H., De Las Salas A., Martin T., Doepke M.

Journal of Innovations in Cardiac Rhythm Management. 10 (12) (pp 3924-3928), 2019. Date of Publication: 2019.

[Article]

Publisher
MediaSphere Medical LLC

Abstract + My Projects + Annotate

Abstract Reference
Complete Reference

Find Similar
Find Citing Articles

Bibliographic Links
UBC eLink

26. **Toward Actionable Practice Parameters for "Dual Diagnosis": Principles of Assessment and Management for Co-Occurring Psychiatric and Intellectual/Developmental Disability.**

Constantino J.N., Strom S., Bunis M., Nadler C., Rodgers T., LePage J., Cahalan C., Stockreef A., Evans L., Jones R., Wilson A.

Current Psychiatry Reports. 22 (2) (no pagination), 2020. Article Number: 9. Date of Publication: 01 Feb 2020.

[Review]

Publisher
Springer

Abstract + My Projects + Annotate

Abstract Reference
Complete Reference

Find Similar
Find Citing Articles

Bibliographic Links
UBC eLink

27. **Prenatal opioid exposure: The next neonatal neuroinflammatory disease.**

Jantzie L.L., Maxwell J.R., Newville J.C., Yellowhair T.R., Kitase Y., Madurai N., Ramachandra S., Bakhireva L.N., Northington F.J., Gerner G., Tekes A., Milio L.A., Brigman J.L., Robinson S., Allan A.

Brain, Behavior, and Immunity. 84 (pp 45-58), 2020. Date of Publication: February 2020.

[Article]

Publisher
Academic Press Inc. (E-mail: apjcs@harcourt.com)

Abstract + My Projects + Annotate

Abstract Reference
Complete Reference

Find Similar
Find Citing Articles

Bibliographic Links
UBC eLink

28. **Outpatient physical therapy for functional neurological disorder: A preliminary feasibility and naturalistic outcome study in a U.S. cohort.**

Maggio J.B., Ospina J.P., Callahan J., Hunt A.L., Stephen C.D., Perez D.L.

Journal of Neuropsychiatry and Clinical Neurosciences. 32 (1) (pp 85-89), 2020. Date of Publication: 2020.

[Article]

Publisher
American Psychiatric Association

Abstract + My Projects + Annotate

Abstract Reference
Complete Reference

Find Similar
Find Citing Articles

Bibliographic Links
UBC eLink

29. **Living with a well-known stranger: Voices of family members to older persons with frontotemporal dementia.**

Tyrrell M., Fossum B., Skovdahl K., Religa D., Hilleras P.

International journal of older people nursing. 15 (1) (pp e12264), 2020. Date of Publication: 01 Mar 2020.

[Article]

Publisher
NLM (Medline)

Abstract + My Projects + Annotate

Abstract Reference
Complete Reference

Find Similar
Find Citing Articles

Bibliographic Links
UBC eLink

30. **Unmet needs for treatment in 102 individuals with brief and limited intermittent psychotic symptoms (BLIPS): implications for current clinical recommendations.**

Fusar-Poli P., De Micheli A., Chalambrides M., Singh A., Augusto C., McGuire P.

Epidemiology and psychiatric sciences. 29 (pp e67), 2019. Date of Publication: 19 Nov 2019.

[Article]

Publisher
NLM (Medline)

Abstract + My Projects + Annotate

Abstract Reference
Complete Reference

Find Similar
Find Citing Articles

Bibliographic Links
UBC eLink

31. **Safety of esophagogastroduodenoscopy in patients with active cocaine use: A pilot study.**

Nguyen A., Desai P.M., Yap J., Yu J., Kotwal V., Attar B.M.

American Journal of Gastroenterology. Conference: 2019 Annual Scientific Meeting and Postgraduate Course of the American College of Gastroenterology, ACG 2019. United States. 114 (Supplement) (pp S330), 2019. Date of Publication: October 2019.

[Conference Abstract]

Publisher
Wolters Kluwer Health

Abstract Article as PDF (161KB) + My Projects
+ Annotate

Ovid Full Text
Abstract Reference
Complete Reference

Find Similar
Find Citing Articles

Bibliographic Links
UBC eLink

32. **Assessment and management of cough among patients with lung cancer in a radiotherapy department in China: A best practice implementation project.**

Zhang L., Wu Y., Du M., He L., Xie G., Wang H., Zhou C., Chen P.

JBI Database of Systematic Reviews and Implementation Reports. 17 (11) (pp 2390-2400), 2019. Date of Publication: 01 Nov 2019.

[Article]

Publisher
Lippincott Williams and Wilkins (E-mail: kathiest.clai@apta.org)

Abstract | Article as PDF (574KB) | + My Projects | + Annotate

Ovid Full Text
Abstract Reference
Complete Reference

Find Similar
Find Citing Articles

Bibliographic Links
UBC eLink

---

33. **The relationship between medication-related problems and behavioural health condition among patients served by a health care for the homeless centre.**

Marks S.A., Moczygemba L.R., Gatewood S.B., Osborn R.D., Wallace N., Lakhani S., Matzke G.R., Goode J.-V.R.

Journal of Pharmaceutical Health Services Research. 3 (3) (pp 173-178), 2012. Date of Publication: September 2012.

[Article]

Publisher
Blackwell Publishing Ltd

Abstract | + My Projects | + Annotate

Abstract Reference
Complete Reference

Find Similar
Find Citing Articles

Bibliographic Links
UBC eLink

---

34. **Development and initial reliability and validity of a new measure of distorted maternal representations: The Mother-Infant Relationship Scale.**

Newman-Morris V., Gray K.M., Simpson K., Newman L.K.

Infant Mental Health Journal. 41 (1) (pp 40-55), 2020. Date of Publication: 01 Jan 2020.

[Article]

Publisher
John Wiley and Sons Inc. (P.O.Box 18667, Newark NJ 07191-8667, United States)

Abstract | + My Projects | + Annotate

Abstract Reference
Complete Reference

Find Similar
Find Citing Articles

Bibliographic Links
UBC eLink

---

35. **Matrix factorization with heterogeneous multiclass preference context.**

Lin J., Pan W., Li L., Chen Z., Ming Z.

Neurocomputing. (no pagination), 2020. Date of Publication: 2020.

[Article]

Publisher
Elsevier B.V.

Abstract | + My Projects | + Annotate

Abstract Reference
Complete Reference

Find Similar
Find Citing Articles

UBC eLink

---

36. **Evaluation of defecation disorders in children with autism spectrum**

disorder by colonic manometry.

Coe A., Ciricillo J., El-Chammas K., Santucci N., Damrongmanee A., Fei L., Liu C., Kaul A.

Journal of Pediatric Gastroenterology and Nutrition. Conference: North American Society for Pediatric Gastroenterology, Hepatology and Nutrition Annual Meeting, NASPGHAN 2019. United States. 69 (Supplement 2) (no pagination), 2019. Date of Publication: November 2019.

[Conference Abstract]

Publisher
Lippincott Williams and Wilkins

▶ Abstract + My Projects + Annotate

Abstract Reference
Complete Reference
Find Similar
Find Citing Articles
Bibliographic Links
UBC eLink

---

37. **Adverse drug reactions: To be or not to be?.**

Saretta F.

Italian Journal of Pediatrics. Conference: 75th Congress of the Italian Society of Pediatrics. Italy. 45 (Supplement 3) (no pagination), 2019. Date of Publication: 2019.

[Conference Abstract]

Publisher
BioMed Central Ltd.

▶ Abstract + My Projects + Annotate

Abstract Reference
Complete Reference
Find Similar
Find Citing Articles
UBC eLink

---

38. **Schizophrenia and cannabis use disorder dual diagnosis - Clinical vignettes.**

Vasiliu O., Vasile D., Voicu V.A.

Medical Cannabis and Cannabinoids. Conference: 4th International Medical Cannabis Conference, CannX 2019. Israel. 2 (2) (pp 15), 2019. Date of Publication: December 2019.

[Conference Abstract]

Publisher
S. Karger AG

▶ Abstract + My Projects + Annotate

Abstract Reference
Complete Reference
Find Similar
Find Citing Articles
Bibliographic Links
UBC eLink

---

39. **Sexual dysfunction and satisfaction in obsessive compulsive disorder: Protocol for a systematic review and meta-analysis.**

Pozza A., Veale D., Marazziti D., Delgadillo J., Albert U., Grassi G., Prestia D., Dettore D.

Systematic Reviews. 9 (1) (no pagination), 2020. Article Number: 8. Date of Publication: 09 Jan 2020.

[Review]

Publisher
BioMed Central Ltd. (E-mail: info@biomedcentral.com)

▶ Abstract + My Projects + Annotate

Abstract Reference
Complete Reference
Find Similar
Find Citing Articles
Full Text
Bibliographic Links
UBC eLink

---

40. **Conventional Versus Stereotactic Image-guided Pedicle Screw Placement during Posterior Lumbar Fusions: A Retrospective**

Ovid Full Text
Abstract Reference

Propensity Score-matched Study of a National Longitudinal Database.

Pendharkar A.V., Rezaii P.G., Ho A.L., Sussman E.S., Veeravagu A., Ratliff J.K., Desai A.M.

Spine. 44 (21) (pp E1272-E1280), 2019. Date of Publication: 01 Nov 2019.

[Article]

Publisher
Lippincott Williams and Wilkins (E-mail: kathiest.clai@apta.org)

Abstract Article as PDF (983KB) + My Projects
+ Annotate

Complete Reference

Find Similar
Find Citing Articles

Bibliographic Links
UBC eLink

---

41. **P.303 Treatment outcomes in patients with prescription narcotic use disorder (TAPE).**

Lindmark S., Westman J., Franck J.

European Neuropsychopharmacology. Conference: ECNP Workshop for Early Career Scientists in Europe 2020. France. 31 (Supplement 1) (pp S48), 2020. Date of Publication: February 2020.

[Conference Abstract]

Publisher
Elsevier B.V.

Abstract + My Projects + Annotate

Abstract Reference
Complete Reference

Find Similar
Find Citing Articles

Bibliographic Links
UBC eLink

---

42. **Not Just for Addiction: The Palliative Clinician's Guide to Using Buprenorphine for Pain (FR458).**

Case A.A., Davis M.P., Anwar S., Hansen E., Kullgren J., Pedraza S.L.

Journal of Pain and Symptom Management. Conference: The Annual Assembly of Hospice and Palliative Care: Education Schedule. United States. 59 (2) (pp 476), 2020. Date of Publication: February 2020.

[Conference Abstract]

Publisher
Elsevier Inc.

Abstract + My Projects + Annotate

Abstract Reference
Complete Reference

Find Similar
Find Citing Articles

Bibliographic Links
UBC eLink

---

43. **New Evidence-Based Guidelines for Dementia Palliative Care (FR418).**

Harrison K.L., Ritchie C.S., Brody A.A., Garrett S.B., Bernstein A., Rosa T., Perez-Cerpa B., Naasan G.

Journal of Pain and Symptom Management. Conference: The Annual Assembly of Hospice and Palliative Care: Education Schedule. United States. 59 (2) (pp 456), 2020. Date of Publication: February 2020.

[Conference Abstract]

Publisher
Elsevier Inc.

Abstract + My Projects + Annotate

Abstract Reference
Complete Reference

Find Similar
Find Citing Articles

Bibliographic Links
UBC eLink

---

44. **Healing Moral Injury of Seriously Ill Vietnam Veterans: A Veterans**

Abstract Reference

Affairs (VA) and National Hospice Palliative Care Organization (NHPCO) Collaboration (FR456).

Blackstone K., Dinescu A., Currie-Gill B., Ramsey-Lucas C.A., Wilson M.S., Laramie J.A., Morgan R., Watson P.

Journal of Pain and Symptom Management. Conference: The Annual Assembly of Hospice and Palliative Care: Education Schedule. United States. 59 (2) (pp 475), 2020. Date of Publication: February 2020.

[Conference Abstract]

Publisher
Elsevier Inc.

▶ Abstract + My Projects + Annotate

Complete Reference

Find Similar
Find Citing Articles

Bibliographic Links
UBC eLink

---

45. **Predictors of NIV treatment in patients with COPD exacerbation complicated by respiratory acidaemia.**

Echevarria C., Steer J., Bourke S.C.

Thorax. Conference: British Thoracic Society Winter Meeting, BTS 2019. United Kingdom. 74 (Supplement 2) (pp A38), 2019. Date of Publication: December 2019.

[Conference Abstract]

Publisher
BMJ Publishing Group

▶ Abstract + My Projects + Annotate

Abstract Reference
Complete Reference

Find Similar
Find Citing Articles

Bibliographic Links
UBC eLink

---

46. **Tailored, psychological intervention for anxiety or depression in people with chronic obstructive pulmonary disease (COPD), TANDEM (Tailored intervention for ANxiety and DEpression Management in COPD): Protocol for a randomised controlled trial.**

Sohanpal R., Pinnock H., Steed L., Heslop Marshall K., Chan C., Kelly M., Priebe S., Roberts C.M., Singh S., Smuk M., Saqi-Waseem S., Healey A., Underwood M., White P., Warburton C., Taylor S.J.C.

Trials. 21 (1) (no pagination), 2020. Article Number: 18. Date of Publication: 06 Jan 2020.

[Article]

Publisher
BioMed Central Ltd. (E-mail: info@biomedcentral.com)

▶ Abstract + My Projects + Annotate

Abstract Reference
Complete Reference

Find Similar
Find Citing Articles

Full Text
Bibliographic Links
UBC eLink

---

47. **Sequential Ipilimumab after Chemoradiotherapy in Curative-Intent Treatment of Patients with Node-Positive Cervical Cancer.**

Mayadev J.S., Enserro D., Lin Y.G., Da Silva D.M., Lankes H.A., Aghajanian C., Ghamande S., Moore K.N., Kennedy V.A., Fracasso P.M., Schilder R.J.

JAMA Oncology. 6 (1) (pp 92-99), 2020. Date of Publication: January 2020.

[Article]

Publisher
American Medical Association (E-mail: smcleod@itsa.ucsf.edu)

▶ Abstract + My Projects + Annotate

Abstract Reference
Complete Reference

Find Similar
Find Citing Articles

Bibliographic Links
UBC eLink

48. **Rate of couple HIV testing in a prenatal care clinic and factors associated with refusal of testing among male partners.**

Pollahan A., Thinkhamrop J., Kongwattanakul K., Chaiyarach S., Sutthasri N., Lao-Unka K.

HIV/AIDS - Research and Palliative Care. 11 (pp 369-375), 2019. Date of Publication: 2019.

[Article]

Publisher
Dove Medical Press Ltd. (PO Box 300-008, Albany, Auckland, New Zealand)

Abstract + My Projects + Annotate

Abstract Reference
Complete Reference
Find Similar
Find Citing Articles
Bibliographic Links
UBC eLink

---

49. **Recognizing ADHD in adults with comorbid mood disorders: Implications for identification and management.**

Goodman D.W., Thase M.E.

Postgraduate Medicine. 121 (5) (pp 31-41), 2009. Date of Publication: 2009.

[Article]

Publisher
Medquest Communications LLC

Abstract + My Projects + Annotate

Abstract Reference
Complete Reference
Find Similar
Find Citing Articles
Bibliographic Links
UBC eLink

---

50. **Public policy state on co-occuring addictive and psychiatric disorders.**

Anonymous

Journal of Addictive Diseases. 20 (3) (pp 121-127), 2001. Date of Publication: 2001.

[Short Survey]

Publisher
Haworth Press Inc.

+ My Projects + Annotate

Complete Reference
Find Similar
Find Citing Articles
Bibliographic Links
UBC eLink

---

51. **Gabapentin drug misuse signals: A pharmacovigilance assessment using the FDA adverse event reporting system.**

Vickers-Smith R., Sun J., Charnigo R.J., Lofwall M.R., Walsh S.L., Havens J.R.

Drug and Alcohol Dependence. 206 (no pagination), 2020. Article Number: 107709. Date of Publication: 1 January 2020.

[Article]

Publisher
Elsevier Ireland Ltd

Abstract + My Projects + Annotate

Abstract Reference
Complete Reference
Find Similar
Find Citing Articles
Bibliographic Links
UBC eLink

---

52. **Series overview and introduction.**

Alexander J.L., Dennerstein L.

Expert review of neurotherapeutics. 7 (11 Suppl) (pp S3-S6), 2007. Date of Publication: Nov 2007.

Abstract Reference
Complete Reference
Find Similar

[Review]

Abstract + My Projects + Annotate

Find Citing Articles

Bibliographic Links
UBC eLink

53. **Opinions of palliative care patients and nurses regarding dignified care.**

Eskigulek Y., Kav S.

Annals of Oncology. Conference: 44th Congress of European Society for Medical Oncology, ESMO 2019. Spain. 30 (Supplement 5) (pp v822), 2019. Date of Publication: October 2019.

[Conference Abstract]

Publisher
Oxford University Press

Abstract + My Projects + Annotate

Abstract Reference
Complete Reference

Find Similar
Find Citing Articles

Bibliographic Links
UBC eLink

54. **SOAR study: New approaches to managing social skills deficits in Turner Syndrome.**

Wolstencroft J., Kerry E., Denyer H., Watkins A., Mandy W., Skuse D.

Hormone Research in Paediatrics. Conference: 58th Annual Meeting of the European Society for Paediatric Endocrinology, ESPE 2019. Austria. 91 (Supplement 1) (pp 315-316), 2019. Date of Publication: September 2019.

[Conference Abstract]

Publisher
S. Karger AG

Abstract + My Projects + Annotate

Abstract Reference
Complete Reference

Find Similar
Find Citing Articles

Bibliographic Links
UBC eLink

55. **Integrating palliative care services in oncology clinics: An application of the Edmonton Symptom Assessment System (ESAS).**

Rauenzahn S.L., Schmidt S., Jones J., Aduba I., Tenner L.L.N.

Journal of Clinical Oncology. Conference: Palliative Care in Oncology Symposium. United States. 34 (26 Supplement 1) (pp 168), 2019. Date of Publication: October 2019.

[Conference Abstract]

Publisher
American Society of Clinical Oncology

Abstract + My Projects + Annotate

Abstract Reference
Complete Reference

Find Similar
Find Citing Articles

Bibliographic Links
UBC eLink

56. **The cascade of care for opioid use disorder: a retrospective study in British Columbia, Canada.**

Piske M., Zhou C., Min J.E., Hongdilokkul N., Pearce L.A., Homayra F., Socias E., McGowan G., Nosyk B.

Addiction (Abingdon, England). (no pagination), 2020. Date of Publication: 03 Jan 2020.

[Article]

Publisher

Abstract Reference
Complete Reference

Find Similar
Find Citing Articles

UBC eLink

NLM (Medline)

▶ Abstract + My Projects + Annotate

57. **Pulmonary vs. extra-pulmonary tuberculosis hospitalizations in the US [1998-2014].**

Banta J.E., Ani C., Bvute K.M., Lloren J.I.C., Darnell T.A.

Journal of Infection and Public Health. 13 (1) (pp 131-139), 2020. Date of Publication: January 2020.

[Article]

Publisher
Elsevier Ltd

▶ Abstract + My Projects + Annotate

Abstract Reference
Complete Reference

Find Similar
Find Citing Articles

Bibliographic Links
UBC eLink

58. **Treatment of cholestatic pruritus in children.**

Cies J.J., Giamalis J.N.

American Journal of Health-System Pharmacy. 64 (11) (pp 1157-1162), 2007. Date of Publication: 01 Jun 2007.

[Review]

Publisher
American Society of Health-Systems Pharmacy

▶ Abstract + My Projects + Annotate

Abstract Reference
Complete Reference

Find Similar
Find Citing Articles

Bibliographic Links
UBC eLink

59. **Aldosterone antagonists in the treatment of heart failure.**

Marcy T.R., Ripley T.L.

American Journal of Health-System Pharmacy. 63 (1) (pp 49-58), 2006. Date of Publication: 01 Jan 2006.

[Review]

Publisher
American Society of Health-Systems Pharmacy

▶ Abstract + My Projects + Annotate

Abstract Reference
Complete Reference

Find Similar
Find Citing Articles

Bibliographic Links
UBC eLink

60. **Pharmacologic options for the treatment of obesity.**

Campbell M.L., Mathys M.L.

American Journal of Health-System Pharmacy. 58 (14) (pp 1301-1308), 2001. Date of Publication: 15 Jul 2001.

[Review]

Publisher
American Society of Health-Systems Pharmacy

▶ Abstract + My Projects + Annotate

Abstract Reference
Complete Reference

Find Similar
Find Citing Articles

Bibliographic Links
UBC eLink

61. **Interval brain imaging for adults with cerebral glioma.**

Thompson G., Lawrie T.A., Kernohan A., Jenkinson M.D.

Abstract Reference
Complete Reference

Cochrane Database of Systematic Reviews. 2019 (12) (no pagination), 2019. Article Number: CD013137. Date of Publication: 24 Dec 2019.

[Article]

Publisher
John Wiley and Sons Ltd (Southern Gate, Chichester, West Sussex PO19 8SQ, United Kingdom. E-mail: vgorayska@wiley.com)

▶ Abstract + My Projects + Annotate

Find Similar
Find Citing Articles

Bibliographic Links
UBC eLink

---

62. **Using machine learning to explain the heterogeneity of schizophrenia. Realizing the promise and avoiding the hype.**

Tandon N., Tandon R.

Schizophrenia Research. 214 (pp 70-75), 2019. Date of Publication: December 2019.

[Article]

Publisher
Elsevier B.V.

▶ Abstract + My Projects + Annotate

Abstract Reference
Complete Reference

Find Similar
Find Citing Articles

Bibliographic Links
UBC eLink

---

63. **Feasibility of Computerized Cognitive-Behavioral Therapy Combined With Bifrontal Transcranial Direct Current Stimulation for Treatment of Major Depression.**

Welch E.S., Weigand A., Hooker J.E., Philip N.S., Tyrka A.R., Press D.Z., Carpenter L.L.

Neuromodulation. 22 (8) (pp 898-903), 2019. Date of Publication: 01 Dec 2019.

[Article]

Publisher
Blackwell Publishing Inc. (E-mail: subscrip@blackwellpub.com)

▶ Abstract + My Projects + Annotate

Abstract Reference
Complete Reference

Find Similar
Find Citing Articles

Bibliographic Links
UBC eLink

---

64. **Right atrial thrombus associated with concurrent myocardial infarction and pulmonary embolism.**

Ali M., Ibrahim A., Kyaw P., Kulkarni A., Siddique M.

American Journal of Respiratory and Critical Care Medicine. Conference: 2019 International Conference of the American Thoracic Society , ATS 2019. United States. 199 (9) (no pagination), 2019. Date of Publication: May 2019.

[Conference Abstract]

Publisher
American Thoracic Society

▶ Abstract + My Projects + Annotate

Abstract Reference
Complete Reference

Find Similar
Find Citing Articles

UBC eLink

---

65. **Understanding Experiences of Social Support as Coping Resources among Immigrant and Refugee Women with Postpartum Depression: An Integrative Literature Review.**

Kassam S.

Issues in mental health nursing. 40 (12) (pp 999-1011), 2019. Date of Publication: 01 Dec 2019.

Abstract Reference
Complete Reference

Find Similar
Find Citing Articles

[Article]

Publisher
NLM (Medline)

Abstract + My Projects + Annotate

Bibliographic Links
UBC eLink

66. **An observational study of the prescribing practices and patient reported outcomes measures in older people with myelodysplasic syndrome.**

Molga A., Wall M., Chhetri R., Wee A., Singhal D., Giri P., To T., Hiwase D.K.

Blood. Conference: 61st Annual Meeting of the American Society of Hematology, ASH 2019. United States. 134 (Supplement 1) (no pagination), 2019. Date of Publication: November 2019.

[Conference Abstract]

Publisher
American Society of Hematology

Abstract + My Projects + Annotate

Abstract Reference
Complete Reference
Find Similar
Find Citing Articles
Bibliographic Links
UBC eLink

67. **Phase II study of midostaurin + chemotherapy in pediatric patients with untreated, newly diagnosed, FLT3-mutated acute myeloid leukemia (AML).**

Reinhardt D., Zwaan C.M., Hoenekopp A., Niolat J., Ifrah S., Noel-Baron F., Locatelli F.

Blood. Conference: 61st Annual Meeting of the American Society of Hematology, ASH 2019. United States. 134 (Supplement 1) (no pagination), 2019. Date of Publication: November 2019.

[Conference Abstract]

Publisher
American Society of Hematology

Abstract + My Projects + Annotate

Abstract Reference
Complete Reference
Find Similar
Find Citing Articles
Bibliographic Links
UBC eLink

68. **Desideromastica: Tactile chew cravings in iron deficiency anemia.**

Scheckel C.J., Acik D.Y., Ashrani A.A., Hook C.C., Kluck L.A., Marshall A.L., Pruthi R.K., Ravindran A., Shah M.V., Wolanskyj A., Go R.S.

Blood. Conference: 61st Annual Meeting of the American Society of Hematology, ASH 2019. United States. 134 (Supplement 1) (no pagination), 2019. Date of Publication: November 2019.

[Conference Abstract]

Publisher
American Society of Hematology

Abstract + My Projects + Annotate

Abstract Reference
Complete Reference
Find Similar
Find Citing Articles
Bibliographic Links
UBC eLink

69. **Diagnosing absolute iron-deficiency anemia in patients on hemodialysis in a tertiary care centre: A retrospective chart review.**

Clayden R.C., Hopman W., MacLeod F., Good D., Garland J., Hookey L., Hay A.E.

Blood. Conference: 61st Annual Meeting of the American Society of

Abstract Reference
Complete Reference
Find Similar
Find Citing Articles

Hematology, ASH 2019. United States. 134 (Supplement 1) (no pagination), 2019. Date of Publication: November 2019.

[Conference Abstract]

Publisher

American Society of Hematology

▶ Abstract + My Projects + Annotate

Bibliographic Links

UBC eLink

---

70. **Collaborating to improve diagnosis and management of children & adolescents with attention deficit hyperactivity disorder.**

Liu D., Wuthrich A., Norlin C.

Pediatrics. Conference: National Conference on Education 2016. United States. 141 (1) (no pagination), 2018. Date of Publication: January 2018.

[Conference Abstract]

Publisher

American Academy of Pediatrics

▶ Abstract + My Projects + Annotate

Abstract Reference
Complete Reference

Find Similar
Find Citing Articles

UBC eLink

---

71. **Rate of postoperative return visits on adenotonsillectomy patients at age of 3.**

Shah S.V., Jensen D., Nicklaus P.

Pediatrics. Conference: National Conference on Education 2016. United States. 141 (1) (no pagination), 2018. Date of Publication: January 2018.

[Conference Abstract]

Publisher

American Academy of Pediatrics

▶ Abstract + My Projects + Annotate

Abstract Reference
Complete Reference

Find Similar
Find Citing Articles

UBC eLink

---

72. **Symptom Clusters and Quality of Life in Hospice Patients with Cancer.**

Omran S., Khader Y., McMillan S.

Asian Pacific journal of cancer prevention : APJCP. 18 (9) (pp 2387-2393), 2017. Date of Publication: 27 Sep 2017.

[Article]

Publisher

NLM (Medline)

▶ Abstract + My Projects + Annotate

Abstract Reference
Complete Reference

Find Similar
Find Citing Articles

Full Text
Bibliographic Links
UBC eLink

---

73. **Dual diagnosis: An intriguing and actual nosographic issue too long neglected.**

Vitali M., Sorbo F., Mistretta M., Scalese B., Porrari R., Galli D., Rotondo C., Solombrino S., Luisa Attilia M., Addolorato G., Aliotta V., Alessandrini G., Attilia F., Barletta G., Battaglia E., Battagliese G., Capriglione I., Carito V., Casciani O., Casella P., Ceccanti M., Cesarini F., Cibin M., Ciccarelli R., Ciolli P., Coriale G., Di Prinzio A., Fagetti R., Falconi E., Federico M., Ferraguti G., Fiore M., Fiorentino D., Gencarelli S., Giuliani A., Greco A., Iannuzzi S., Intaschi G., Janiri L., Lagrutta A., La Torre G., Laviola G., Ledda R., Leggio L., Leonardi C.,

Abstract Reference
Complete Reference

Find Similar
Find Citing Articles

Bibliographic Links
UBC eLink

Loffreda A., Lugoboni F., Macri S., Mancinelli R., Marconi M., Maremmani I., Maviglia M., Messina M.P., Montesano F., Pascale E., Parisi M., Perciballi R., Pisciotta F., Spinnato G., Valchera A., Zavan V.

Rivista di Psichiatria. 53 (3) (pp 154-159), 2018. Date of Publication: May-June 2018.

[Review]

Publisher
Il Pensiero Scientifico Editore s.r.l. (Via Giovanni Valdarno 8, Roma 00138, Italy)

▶ Abstract + My Projects + Annotate

---

74. **Angiotensin II: A New Vasopressor for the Treatment of Distributive Shock.**

Allen J.M., Gilbert B.W.

Clinical Therapeutics. 41 (12) (pp 2594-2610), 2019. Date of Publication: December 2019.

[Review]

Publisher
Excerpta Medica Inc.

▶ Abstract + My Projects + Annotate

Abstract Reference
Complete Reference

Find Similar
Find Citing Articles

Bibliographic Links
UBC eLink

---

75. **P.321 Clinical parameters associated with augmentation and combination treatment in unipolar depression.**

Bartova L., Dold M., Mendlewicz J., Souery D., Serretti A., Porcelli S., Zohar J., Montgomery S., Kasper S.

European Neuropsychopharmacology. Conference: 32nd ECNP Congress. Denmark. 29 (Supplement 6) (pp S229-S230), 2019. Date of Publication: December 2019.

[Conference Abstract]

Publisher
Elsevier B.V.

▶ Abstract + My Projects + Annotate

Abstract Reference
Complete Reference

Find Similar
Find Citing Articles

Bibliographic Links
UBC eLink

---

76. **Managing Patients With Severe and Enduring Anorexia Nervosa: When Is Enough, Enough?.**

Yager J.

The Journal of nervous and mental disease. (no pagination), 2019. Date of Publication: 10 Dec 2019.

[Article]

Publisher
NLM (Medline)

▶ Abstract + My Projects + Annotate

Abstract Reference
Complete Reference

Find Similar
Find Citing Articles

UBC eLink

---

77. **The role of sleep in assessing and treating psychiatric conditions.**

Appavoo S., Chirwa S.

Sleep Medicine. Conference: 15th World Sleep Congress. Canada. 64 (Supplement 1) (pp S16), 2019. Date of Publication: December 2019.

Abstract Reference
Complete Reference

[Conference Abstract]

Publisher
Elsevier B.V.

Abstract + My Projects + Annotate

Find Similar
Find Citing Articles

Bibliographic Links
UBC eLink

---

78. **C.02.02 Managing ADHD and comorbid mood disorders.**

Goodman D.

European Neuropsychopharmacology. Conference: 32nd ECNP Congress. Denmark. 29 (Supplement 6) (pp S591), 2019. Date of Publication: December 2019.

[Conference Abstract]

Publisher
Elsevier B.V.

Abstract + My Projects + Annotate

Abstract Reference
Complete Reference

Find Similar
Find Citing Articles

Bibliographic Links
UBC eLink

---

79. **Children's bullying involvement and maternal depressive symptoms.**

Nomaguchi K., Fettro M.N.

Social Science and Medicine. 245 (no pagination), 2020. Article Number: 112695. Date of Publication: January 2020.

[Article]

Publisher
Elsevier Ltd

Abstract + My Projects + Annotate

Abstract Reference
Complete Reference

Find Similar
Find Citing Articles

Bibliographic Links
UBC eLink

---

80. **Patients' Experience of Living with a Small Abdominal Aortic Aneurysm and its Effect on Health Related Quality of Life While Being Under Surveillance: A Systematic Review and Synthesis.**

Lyttkens L., Wanhainen A., Svensjo S., Hultgren R., Bjork M., Jangland E.

European Journal of Vascular and Endovascular Surgery. Conference: The European Society for Vascular Surgery 32nd Annual Meeting 2018. Spain. 58 (6 Supplement 2) (pp e370-e372), 2019. Date of Publication: December 2019.

[Conference Abstract]

Publisher
W.B. Saunders Ltd

Abstract + My Projects + Annotate

Abstract Reference
Complete Reference

Find Similar
Find Citing Articles

Bibliographic Links
UBC eLink

---

81. **Near-infrared spectroscopy (NIRS) in the evaluation of psychogenic pseudosyncope - Moving towards a simplified diagnostic pathway.**

Claffey P., Perez-Denia L., Rivasi G., Ungar A., Finucane C., Kenny R.A.

European Heart Journal. Conference: European Society of Cardiology Congress, ESC 2019. France. 40 (Supplement 1) (pp 3548), 2019. Date of Publication: October 2019.

[Conference Abstract]

Publisher
Oxford University Press

Abstract Reference
Complete Reference

Find Similar
Find Citing Articles

Bibliographic Links
UBC eLink

Abstract + My Projects + Annotate

82. **Struma ovarii with atypical features and synchronous primary thyroid cancer: a case report and review of the literature.**

Siegel M.R., Wolsky R.J., Alvarez E.A., Mengesha B.M.

Archives of Gynecology and Obstetrics. 300 (6) (pp 1693-1707), 2019. Date of Publication: 01 Dec 2019.

[Article]

Publisher
Springer

Abstract + My Projects + Annotate

Abstract Reference
Complete Reference
Find Similar
Find Citing Articles
Bibliographic Links
UBC eLink

83. **Friend or foe: Food-dependent exercise-induced anaphylaxis associated with acute coronary syndrome aggravated by adrenaline and aspirin: A case report.**

Toya T., Kagami K., Adachi T.

European Heart Journal - Case Reports. 3 (3) (no pagination), 2019. Article Number: ytz143. Date of Publication: 17 Sep 2019.

[Article]

Publisher
Oxford University Press

Abstract + My Projects + Annotate

Abstract Reference
Complete Reference
Find Similar
Find Citing Articles
Full Text
Bibliographic Links
UBC eLink

84. **Unmet needs in treatment of depression: Comorbid anxiety and management.** Depresyon tedavisinde karsilanmamis ihtiyaclar: Estanili anksiyete ve yaklasim **<Depresyon tedavisinde karsilanmamis ihtiyaclar: Estanili anksiyete ve yaklasim.>**

Dilbaz N., Darcin A.E., Cavus S.Y.

Klinik Psikofarmakoloji Bulteni. 21 (SUPPL. 1) (pp S10-S19), 2011. Date of Publication: 2011.

[Review]

Publisher
Cukurova Univ Tip Fakultesi Psikiyatri Anabilim Dali

Abstract + My Projects + Annotate

Abstract Reference
Complete Reference
Find Similar
Find Citing Articles
Bibliographic Links
UBC eLink

85. **Substance use disorder in individuals with mild intellectual disabilities; collaboration needed.** Verslaving bij mensen met een lichte verstandelijke beperking; versterk de zorgketen **<Verslaving bij mensen met een lichte verstandelijke beperking; versterk de zorgketen.>**

Van Der Nagel J.E.L., Van Duijvenbode N., Van Horsen S.W.L.

Tijdschrift voor Psychiatrie. 6 (11) (pp 798-803), 2019. Date of Publication: 2019.

[Article]

Publisher
Uitgeverij Boom (E-mail: desk@boomonderwijs.nl)

Abstract + My Projects + Annotate

Abstract Reference
Complete Reference
Find Similar
Find Citing Articles
Bibliographic Links
UBC eLink

86. **Opioid-related diagnoses and concurrent claims for HIV, HBV, or HCV among medicare beneficiaries, United States, 2015.**

Chang M.-H., Moonesinghe R., Schieber L.Z., Truman B.I.

Journal of Clinical Medicine. 8 (11) (no pagination), 2019. Article Number: 1768. Date of Publication: November 2019.

[Article]

Publisher
MDPI AG (Postfach, Basel CH-4005, Switzerland. E-mail: indexing@mdpi.com)

▶ Abstract + My Projects + Annotate

Abstract Reference
Complete Reference
Find Similar
Find Citing Articles
Full Text
Bibliographic Links
UBC eLink

87. **Understanding Barriers and Facilitators to the Uptake of Best Practices for the Treatment of Co-Occurring Chronic Pain and Opioid Use Disorder.**

Varley A.L., Lappan S., Jackson J., Goodin B.R., Cherrington A.L., Copes H., Hendricks P.S.

Journal of Dual Diagnosis. (no pagination), 2019. Date of Publication: 2019.

[Article]

Publisher
Routledge (E-mail: aabs@uw.edu)

▶ Abstract + My Projects + Annotate

Abstract Reference
Complete Reference
Find Similar
Find Citing Articles
UBC eLink

88. **Anesthesia management of special patient populations undergoing electroconvulsive therapy: A review.**

Wajima Z.

Journal of Nippon Medical School. 86 (2) (pp 70-80), 2019. Date of Publication: 2019.

[Review]

Publisher
Medical Association of Nippon Medical School (E-mail: jnms@nms.ac.jp)

▶ Abstract + My Projects + Annotate

Abstract Reference
Complete Reference
Find Similar
Find Citing Articles
Bibliographic Links
UBC eLink

89. **Clinical Validity of DSM-5 Attenuated Psychosis Syndrome: Advances in Diagnosis, Prognosis, and Treatment.**

Salazar De Pablo G., Catalan A., Fusar-Poli P.

JAMA Psychiatry. (no pagination), 2019. Date of Publication: 2019.

[Review]

Publisher
American Medical Association (E-mail: smcleod@itsa.ucsf.edu)

▶ Abstract + My Projects + Annotate

Abstract Reference
Complete Reference
Find Similar
Find Citing Articles
UBC eLink

90. **Why we urgently need improved seizure and epilepsy therapies for children and neonates.**

Abstract Reference
Complete Reference

Pressler R., Lagae L.

Neuropharmacology. (pp 107854), 2019. Date of Publication: 18 Nov 2019.

[Review]

Publisher
NLM (Medline)

▶ Abstract + My Projects + Annotate

Find Similar
Find Citing Articles

UBC eLink

---

91. **Representation of people with comorbidity and multimorbidity in clinical trials of novel drug therapies: An individual-level participant data analysis.**

Hanlon P., Hannigan L., Rodriguez-Perez J., Fischbacher C., Welton N.J., Dias S., Mair F.S., Guthrie B., Wild S., McAllister D.A.

BMC Medicine. 17 (1) (no pagination), 2019. Article Number: 201. Date of Publication: 12 Nov 2019.

[Article]

Publisher
BioMed Central Ltd. (E-mail: info@biomedcentral.com)

▶ Abstract + My Projects + Annotate

Abstract Reference
Complete Reference

Find Similar
Find Citing Articles

Full Text
Bibliographic Links
UBC eLink

---

92. **First-line managers' experience of the use of audit and feedback cycle in specialist mental health care: A qualitative case study.**

Pedersen M.S., Landheim A., Moller M., Lien L.

Archives of psychiatric nursing. 33 (6) (pp 103-109), 2019. Date of Publication: 01 Dec 2019.

[Article]

Publisher
NLM (Medline)

▶ Abstract + My Projects + Annotate

Abstract Reference
Complete Reference

Find Similar
Find Citing Articles

Bibliographic Links
UBC eLink

---

93. **How Do Common Comorbidities Modify the Association of Frailty With Survival After Elective Noncardiac Surgery? A Population-Based Cohort Study.**

Hui Y., van Walraven C., McIsaac D.I.

Anesthesia and analgesia. 129 (6) (pp 1699-1706), 2019. Date of Publication: 01 Dec 2019.

[Article]

Publisher
NLM (Medline)

▶ Abstract Article as PDF (293KB) + My Projects
+ Annotate

Ovid Full Text
Abstract Reference
Complete Reference

Find Similar
Find Citing Articles

Bibliographic Links
UBC eLink

---

94. **A systematic review of the integration of palliative care in dementia management.**

Senderovich H., Retnasothie S.

Palliative & supportive care. (pp 1-12), 2019. Date of Publication: 18 Nov 2019.

Abstract Reference
Complete Reference

Find Similar

[Article]

Publisher
NLM (Medline)

Find Citing Articles

UBC eLink

▶ Abstract + My Projects + Annotate

---

95. **Improving quality of antipsychotic polypharmacy: a pilot study.**

Hoilund H., Galea D.P., Ringen P.A.

Nordic Journal of Psychiatry. (no pagination), 2019. Date of Publication: 2019.

[Article]

Publisher
Taylor and Francis Ltd

Abstract Reference
Complete Reference
Find Similar
Find Citing Articles
UBC eLink

▶ Abstract + My Projects + Annotate

---

96. **Implementing a Tablet-Guided Mindfulness Meditation Intervention to Reduce Anxiety in Women Receiving Radiotherapy for Breast Cancer.**

Grewal N.K.S., Arya R., Malik R., Hasan Y., Golden D.W., McCall A.R.R.

International Journal of Radiation Oncology Biology Physics. Conference: The American Society for Radiation Oncology 61st Annual Meeting. United States. 105 (1 Supplement) (pp E588), 2019. Date of Publication: 1 September 2019.

[Conference Abstract]

Publisher
Elsevier Inc.

Abstract Reference
Complete Reference
Find Similar
Find Citing Articles
Bibliographic Links
UBC eLink

▶ Abstract + My Projects + Annotate

---

97. **Clinical and Dosimetric Correlation between Nausea-Vomiting, Fatigue and Late Dysphagia Related to Nervous System Structures during Radical Radiotherapy for Squamous Cell Carcinomas of Head and Neck.**

Basu T., Bhaskar N., Armugam K., Ganeshan P., Saxena U.

International Journal of Radiation Oncology Biology Physics. Conference: The American Society for Radiation Oncology 61st Annual Meeting. United States. 105 (1 Supplement) (pp E410-E411), 2019. Date of Publication: 1 September 2019.

[Conference Abstract]

Publisher
Elsevier Inc.

Abstract Reference
Complete Reference
Find Similar
Find Citing Articles
Bibliographic Links
UBC eLink

▶ Abstract + My Projects + Annotate

---

98. **MGMT-Deficiency Is a Biomarker to Guide Treatment of Solid Tumors with Temozolomide and ATR Inhibitors.**

Jackson C., Noorbakhsh S., Kalathil A.N., Sundaram R.K., Bindra R.S.

International Journal of Radiation Oncology Biology Physics. Conference: The American Society for Radiation Oncology 61st Annual Meeting. United States. 105 (1 Supplement) (pp E638-E639), 2019. Date of Publication: 1 September 2019.

Abstract Reference
Complete Reference
Find Similar
Find Citing Articles

[Conference Abstract]

Publisher
Elsevier Inc.

▶ Abstract + My Projects + Annotate

Bibliographic Links
UBC eLink

---

99. **The Use of Complementary and Integrative Therapies as Adjunct Interventions During Radiotherapy for Patients with Cancer: A Systematic Review.**

Lapen K., Cha E., McArthur M.A., Rosenberg D.M., Rooney M.K., Arya R., Son C.H., McCall A.R.R., Golden D.W.

International Journal of Radiation Oncology Biology Physics. Conference: The American Society for Radiation Oncology 61st Annual Meeting. United States. 105 (1 Supplement) (pp E577-E578), 2019. Date of Publication: 1 September 2019.

[Conference Abstract]

Publisher
Elsevier Inc.

▶ Abstract + My Projects + Annotate

Abstract Reference
Complete Reference

Find Similar
Find Citing Articles

Bibliographic Links
UBC eLink

---

100. **Pharmacotherapies for co-occurring substance use and bipolar disorders: A systematic review.**

Coles A.S., Sasiadek J., George T.P.

Bipolar Disorders. 21 (7) (pp 595-610), 2019. Date of Publication: 01 Nov 2019.

[Review]

Publisher
Blackwell Publishing Inc. (E-mail: subscrip@blackwellpub.com)

▶ Abstract + My Projects + Annotate

Abstract Reference
Complete Reference

Find Similar
Find Citing Articles

Bibliographic Links
UBC eLink

© 2020 Ovid Technologies, Inc. All rights reserved. OvidUI_04.04.00.037, SourceID 7eebd4b
